# Characterization of global and region‐specific transcriptomic alterations in the bladder following acute partial bladder outlet obstruction in female mini‐swine

**DOI:** 10.14814/phy2.71074

**Published:** 2026-08-19

**Authors:** Yi Xi Wu, Gokhan Gundogdu, Madison Rivero, Caila Ruiz‐May, Ahmet Tarik Gundogdu, Victor Pham, Keith Rourke, Zhina Sadeghi, Joel Gelman, Joshua R. Mauney

**Affiliations:** ^1^ Department of Urology University of California, Irvine Orange California USA; ^2^ Department of Biomedical Engineering University of California, Irvine Irvine California USA; ^3^ Department of Biological Sciences University of California, Irvine Irvine California USA

**Keywords:** bladder, dome, obstruction, RNA sequencing, trigone

## Abstract

Partial bladder outlet obstruction (pBOO) is a fibroproliferative disorder that results in poorly compliant, high‐pressure bladders due to lower urinary tract occlusion. The mechanisms governing pBOO‐induced bladder remodeling remain poorly understood. The goals of the present study were to profile global and region‐specific transcriptomes of porcine bladders in response to pBOO using RNA sequencing and to identify genes and pathways differentially affected in the bladder dome and trigone by obstructive injury. Nine adult female Yucatan mini‐swine were randomized and divided into either nonsurgical controls (*n* = 5) or those subjected to pBOO for 4 weeks (*n* = 4). Post‐pBOO swine demonstrated bladder smooth muscle hypertrophy as well as significant declines in bladder capacity (35% ± 7% of baseline) and compliance (38% ± 10% of baseline). The global transcriptome of obstructed bladders revealed enrichment of processes related to loss of urothelial barrier function, immune activation, cell cycle regulation, extracellular matrix organization, and signal transduction similar to human and rodent evaluations of pBOO. Moreover, our data also showed that adaptive responses to pBOO are regionally dependent rather than globally uniform, with zonal‐specific divergence observed across hypoxic, fibrotic, metabolic, and neuronal pathways between the obstructed dome and trigone. These results highlight the regional heterogeneity of bladder responses to obstructive urologic disease.

## 1 INTRODUCTION

Partial bladder outlet obstruction (pBOO) is a highly burdensome and prevalent pathology in men and women, resulting in high‐pressure/low‐flow micturition patterns due to anatomical or functional occlusion of the lower urinary tract (Li et al., 2024; Yande & Joshi, 2011). This condition is common in a number of urologic disorders, including neurogenic bladder and sphincter dysfunction, posterior urethral valves, benign prostatic obstruction/enlargement, severe dysfunctional voiding, urethral stricture disease, and acquired obstruction from iatrogenic injuries (Farrugia, 2016; Komninos & Mitsogiannis, 2014; Patel et al., 2012; Taweel & Seyam, 2015; Tritschler et al., 2013). Over time, pBOO can lead to bladder overdistension, urinary retention, urinary tract infections, and myogenic failure with urinary catheter dependence or alternatively results in diminished capacity with poor compliance from progressive fibroproliferative tissue remodeling (Fusco et al., 2018). Urinary incontinence and renal failure are clinical manifestations of pBOO, which severely impact patient quality of life and contribute substantially to the costs of disease management (Bergamin & Kiosoglous, 2017; Dmochowski, 2005; Perez‐Aizpurua et al., 2024). Current treatment modalities for pBOO include cystoscopic ablation of obstructive masses (Pagano et al., 2015); the use of α‐adrenergic blockers and anti‐cholinergic medications to relax the bladder neck and detrusor (Bade et al., 2025); clean intermittent catheterization to manage urinary retention (Chapple et al., 2024); and, in cases of poorly compliant bladders, enterocystoplasty is performed to preserve renal health (Jeong & Oh, 2020). Despite these interventions, many patients will still deteriorate to an irreversible state of bladder dysfunction (Kim et al., 2021). Therefore, a deeper understanding of the pathophysiologic mechanisms of pBOO is needed to design efficacious and durable interventions to mitigate the disease.

Human and rodent studies of pBOO have shown that modulation of inflammatory and pro‐fibrotic signaling pathways induces histological and functional alterations within the obstructed bladder in three sequential stages: hypertrophy, compensation, and decompensation (Fusco et al., 2018; Komninos & Mitsogiannis, 2014; Levin et al., 2004; Metcalfe et al., 2010). During the hypertrophic phase, bladder overdistension secondary to pBOO restricts blood flow and subsequent blood oxygen saturation within the bladder wall, creating hypoxic conditions in the detrusor (Lin et al., 2011; Scheepe et al., 2011), thereby stimulating de novo angiogenesis and smooth muscle growth to overcome increased resistance to urine flow (Alsaikhan et al., 2015; Fusco et al., 2018; Komninos & Mitsogiannis, 2014; Levin et al., 2004; Metcalfe et al., 2010). Moreover, urothelial hypoxia in obstructed bladders disrupts the blood‐urine barrier by inhibiting expression of tight junction proteins (Luo et al., 2024) and has been significantly correlated with deterioration of bladder function in patients (Radford et al., 2019). Next, subsequent activation of growth suppressor gene expression causes cessation of bladder expansion and mitigation of vascular processes during the compensation phase (Fusco et al., 2018; Komninos & Mitsogiannis, 2014; Levin et al., 2004; Metcalfe et al., 2010). Finally, persistent pBOO leads to a decompensated state in which cyclical ischemia–reperfusion injury during micturition causes progressive smooth muscle loss, excessive deposition of collagenous extracellular matrix (ECM), and neuronal damage, ultimately resulting in organ dysfunction and preventing complete bladder emptying (Fusco et al., 2018; Komninos & Mitsogiannis, 2014; Metcalfe et al., 2010).

Previous assessments of bladder transcriptomic responses to obstructive injury have primarily relied on whole‐organ evaluations in animal models (Duan et al., 2017; Ito et al., 2021; von Siebenthal et al., 2023; Zhu et al., 2022) or focal biopsies of clinical specimens (Akshay et al., 2024; Gheinani et al., 2018) to identify the genes and networks that drive disease progression. However, the bladder is composed of distinct regions, including the dome, defined as the anterosuperior region with boundaries of the apex and fundus, and the trigone, which are structurally different and play divergent functional roles in organ physiology (Sanchez Freire et al., 2011). In particular, the dome is situated at the apex of the bladder and primarily expands to accommodate increasing urine volume, while release of neurotransmitters such as acetylcholine and ATP in this region initiates detrusor contraction and urine expulsion (Andersson & Arner, 2004). On the other hand, the trigone is a triangular zone positioned at the base of the bladder between the ureteral orifices and the internal urethral opening, which supports normal bladder filling by regulating the passage of urine from the distal ureters while preventing reflux (Viana et al., 2007). Compared with the dome, the trigone undergoes minimal deflection during storage and voiding cycles but serves important functions, including pain perception and coordination of micturition via detection of bladder filling (Flores et al., 2026; French & Bhambore, 2011; Lv et al., 2017; Sadahira et al., 2025). In addition, the musculature of the trigone exhibits more spontaneous activity within individual myocytes, contains more connective tissue, and shows differences in gap junction proteins compared with the dome (Sanchez Freire et al., 2011).

Recent large‐scale sequencing analyses of urothelial biopsies from adolescent female patients with micturition dysfunction have revealed significant variation in the urothelial transcriptomes of the bladder dome and trigone, suggesting that selective regional effects can result from urologic pathologies (Zeber‐Lubecka et al., 2022). Given these findings, characterizing the local effects of pBOO on both the bladder dome and trigone may lead to the discovery of novel druggable targets to improve treatment of the disease. Therefore, the goals of the present study were to profile global and region‐specific transcriptomes of full‐thickness porcine bladder specimens in response to acute pBOO using RNA sequencing, and to identify genes and pathways that are differentially affected in the bladder dome and trigone by obstructive injury. We utilized an established model of pBOO in female swine whereby voiding occurs via a urinary catheter equipped with controlled release valves to precisely regulate urinary outlet resistance (Affas et al., 2019). A urinary outlet pressure of 70 ± 15 cmH_2_O was selected for pBOO to achieve detrusor leak point pressures >40 cmH_2_O, which have been clinically correlated with an elevated risk of upper urinary tract damage (McGuire et al., 1981; Snodgrass & Gargollo, 2010). Female swine were deployed as study cohorts given their anatomical and physiological similarities with the human urinary tract (Sloff et al., 2014), large bladder size relative to rodents, enabling regional tissue sampling for multiple analyses, and their wide, patent urethras, which can accommodate standard urologic instrumentation and catheterization, in contrast to their tortuous male counterparts (Affas et al., 2019).

## 2 MATERIALS AND METHODS

### 2.1 Surgical procedures

All surgical interventions, imaging modalities, and animal husbandry procedures were evaluated and approved by the Institutional Animal Care and Use Committee of the University of California, Irvine under protocol AUP‐22‐130. This protocol was also performed in compliance with the ethical considerations expressed in the Declaration of Helsinki and the National Institutes of Health's Guidelines for the Care and Use of Laboratory Animals, as well as ARRIVE guidelines (https://arriveguidelines.org). Nine adult female Yucatan mini‐swine (~4 months of age, 30–40 kg; Premier BioSource, Ramona, CA) were randomized into two experimental groups, including nonsurgical controls (NSC, *n* = 5) and those subjected to pBOO for 4 weeks (*n* = 4) using our previously reported methods summarized below (Affas et al., 2019). All swine were communally housed in individual pens within a temperature controlled (66–68°F, 62%–66% relative humidity) vivarium and given free access to water and food (Labdiet® Mini‐pig HF High Fortification Grower, PMI Nutrition International, LLC, Arden Hills, MN, USA) for the duration of the study.

**FIGURE 1:**
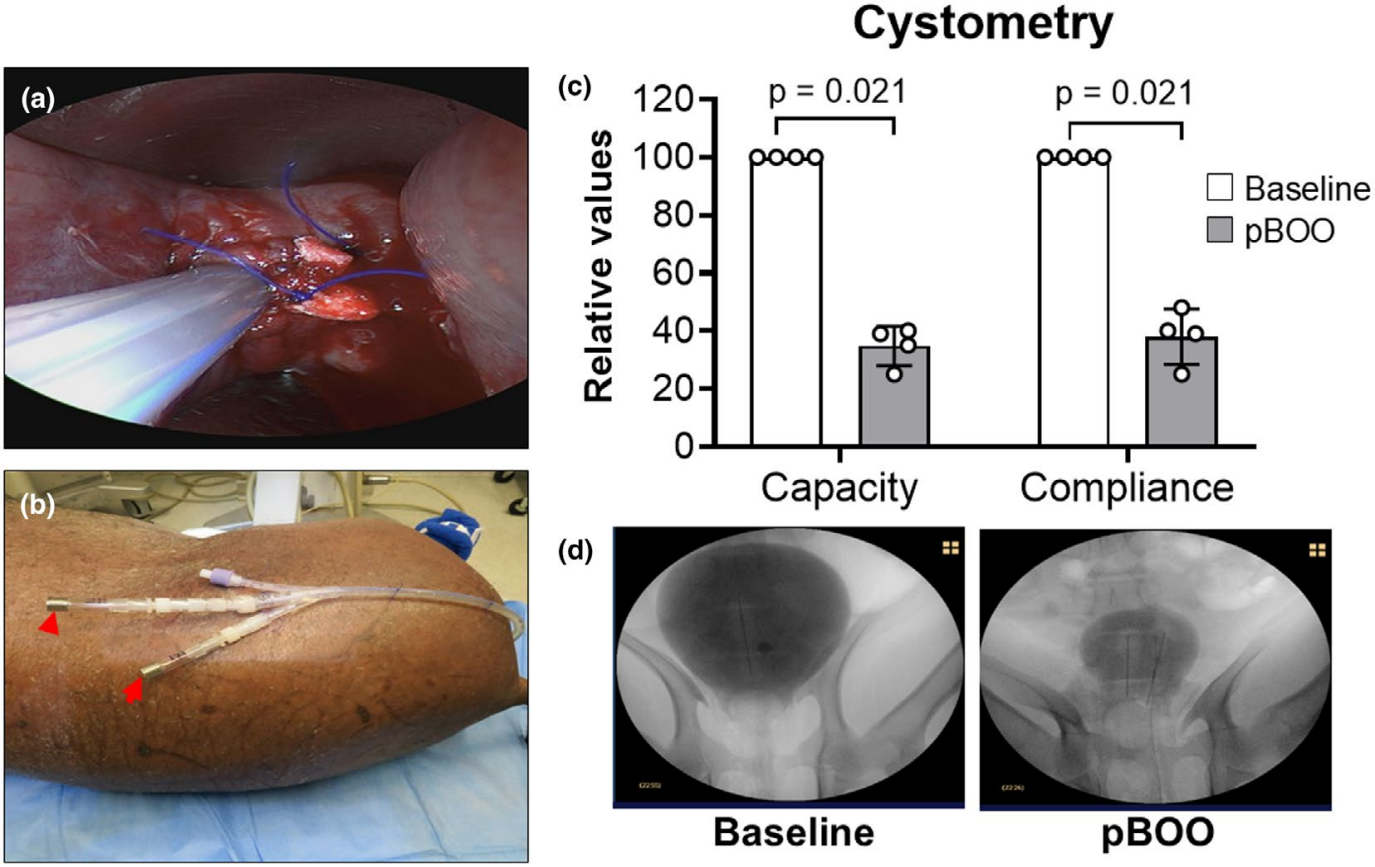
Porcine pBOO model and urodynamic outcomes. (a) Insertion of a Foley catheter into the female porcine urethra and placement of a peri‐urethral purse string to establish a fluid‐tight seal around the urinary meatus to allow for management of urinary outlet resistance. (b) A Foley catheter was mounted on the animal's flank and fitted with controlled‐release valves (red arrowheads) to produce pBOO at a pressure of 70 ± 15 cmH_2_O. (c) Urodynamic analyses of bladder capacity and compliance in swine at baseline and 4 weeks following pBOO. All urodynamic data are displayed relative to baseline values. *n* = 4 animals per data point. *p*‐values reflect comparisons to respective noninjured baselines. Data were analyzed with Mann–Whitney *U* test and displayed as means ± standard deviation. (d) Representative cystograms of swine bladders acquired at 20 cmH_2_O pressure at baseline and 4 weeks following pBOO. pBOO, partial bladder outlet obstruction.

For pBOO model creation, swine were fasted overnight and provided water ad libitum. General anesthesia was induced via intramuscular injection of 2.2 mg/kg Anased (xylazine hydrochloride, Lloyd Inc., IA, USA) and 4.4 mg/kg Telazol (tiletamine and zolazepam, Zoetis Inc., Parsippany, NJ, USA) and maintained with 1%–4% isoflurane via an endotracheal tube. Animals were positioned supine and secured to the table with the hind limbs flexed at the hips. The genital area was disinfected with povidone iodine and 70% ethanol, and the genital confluence was gently retracted bilaterally using a vaginal speculum to access the urethral orifice. A 22‐French, three‐way silicone Foley catheter was then inserted into the bladder, inflated, and baseline cystography and urodynamic evaluations were performed as described below. Next, the urethral orifice was closed around the catheter using two peri‐urethral purse sutures (2–0 polypropylene) to generate a fluid‐tight seal at the urinary meatus (Figure 1a). The catheter was then anchored to the animal's flank via interrupted, nonabsorbable sutures to prevent displacement. Foley catheter outlets were fitted with controlled‐release valves (Lee Company, Westbrook, CT) with a cracking pressure of 70 ± 15 cmH_2_O (Figure 1b) and were maintained for 4 weeks, with a 2‐week replacement to avoid fouling by urinary sediments. Animals were administered a transdermal fentanyl patch (1–4 μg/kg) 24 h prior to surgery and received 1.1 mg/kg intramuscular flunixin meglumine (Banamine®, Merck Animal Health, Rahway, NJ, USA) injections for three consecutive days post‐operatively for pain control. In addition, a 3.9 mg/day oxybutynin patch (Oxytrol, Merck Animal Health, Rahway, NJ, USA) was used to reduce bladder spasms. Animals also received a five‐day course of 7.5 mg/kg enrofloxacin (Baytril, Bayer Healthcare LLC, KA, USA) intramuscularly after surgical manipulations to mitigate potential bacterial infections. Animals subjected to pBOO were monitored daily for pain or distress over the 4‐week study period. All swine were sacrificed with 0.50 mL/kg Euthasol (pentobarbital sodium and phenytoin sodium, Virbac, Westlake, TX).

### 2.2 Cystometry and cystography

Cystometry and contrast cystography were conducted at baseline, prior to surgical manipulations, and at 4 weeks post‐pBOO (*n* = 4 swine per time point) to assess bladder capacity, compliance, and overall bladder anatomy. After establishing urethral access under general anesthesia, Foley catheter ports were individually connected to a urodynamics system (Goby™ CT, Laborie, Portsmouth, NH, USA) to facilitate saline infusion into the bladder and to monitor bladder outlet pressures. In addition, a 12 French catheter was inserted into the rectum and connected to an abdominal pressure transducer to evaluate fluctuations in extra‐vesical pressure. Two or more filling cycles were conducted at 20 mL/min using 37°C saline supplemented with a 1:10 dilution of contrast agent (Omnipaque 300, GE Healthcare, Milwaukee, WI, USA). Bladder infusion was terminated after achieving a filling pressure of 20 cmH_2_O, and a cystogram image was acquired with a C‐arm fluoroscopy (BV Pulsera; Philips, Eindhoven, Netherlands), and bladder capacity was recorded. Bladder compliance was determined by quantifying Δvolume/Δpressure between start and end‐point pressures between filling cycles.

### 2.3 Histological, immunohistochemical, and histomorphometric analyses

Following euthanasia and bladder harvest, full‐thickness dome and trigone specimens [2 pieces (1 cm^2^ each) per region per pig] were excised from NSC (*n* = 5 swine) as well as pBOO groups (*n* = 4 swine) and prepared for standard histological evaluations or RNA isolation procedures described below. Bladder samples were fixed for 24 h in 10% neutral buffered formalin, dehydrated in graded alcohol solutions, and embedded in paraffin. Ten‐micron tissue sections were cut on a microtome, stained with Masson's trichrome, and global images were acquired from tiled 20X fields using an Axioplan‐2 microscope (Carl Zeiss MicroImaging, Thornwood, NY) and Axiovision software (version 4.8). Detrusor smooth muscle content as well as collagen content was evaluated using ImageJ software by quantifying the percentage of red‐ and blue‐stained areas, respectively, in each specimen per total tissue area examined following color segmentation and image thresholding tools as previously described (Affas et al., 2019). For immunohistochemical (IHC) analyses, parallel tissue sections from each group were deparaffinized in xylene, rehydrated in graded alcohol solutions, and subjected to antigen retrieval in 10 mM citrate buffer (pH 6.0). Specimens were then incubated in blocking buffer solution consisting of phosphate‐buffered saline (PBS), 5% fetal bovine serum, 1% fetal bovine serum albumin, and 0.3% Triton X‐100 for 1 h at 20°C. For antigen detection, samples were probed with the following primary antibodies overnight at 4°C including anti‐uroplakin (UPK) 1A (cat. no. NBP2‐79733, mouse IgG, 1:100 dilution, Novus biologicals, Centennial, CO), anti‐pan‐cytokeratin (CK) (cat. no. M3515, mouse IgG, 1:100 dilution, Agilent, Santa Clarita, CA), anti‐hypoxia inducible factor‐1α (HIF‐1ɑ) (cat. no. PA1‐16601, rabbit IgG, 1:100 dilution, Thermo Fisher Scientific, Cambridge, MA), and anti‐vascular endothelial growth factor (VEGF) (cat. no. bs‐0279R, rabbit IgG, 1:100 dilution, Thermo Fisher Scientific). After washing with PBS, specimens were incubated with species‐matched Alexa Fluor647‐conjugated secondary antibodies (mouse: cat. no. A‐3157, 1:200 dilution, Thermo Fisher Scientific; rabbit: cat. no. A‐31573, 1:200 dilution, Thermo Fisher Scientific), counterstained with 4′, 6‐diamidino‐2‐phenyllindole (DAPI), and imaged as described above. Histomorphometric analyses (*n* = 4–5 pigs for each region/group) were performed on global microscopic fields from stained full thickness bladder specimens and relative immunoreactivity was determined by quantifying selective marker expression area in either the detrusor (HIF1ɑ and VEGF) or epithelial (UPK1A and pan‐CK) compartments and normalizing values to total stained area examined using published methods (Affas et al., 2019).

### 2.4 Isolation of total RNA from tissue

Full‐thickness bladder dome and trigone specimens from NSC (*n* = 5 swine) and pBOO groups (*n* = 4 swine) described above were flash‐frozen in liquid nitrogen and stored at −80°C until used. Total RNA was isolated using the RNeasy Plus Kit (cat. no. 74134, Qiagen, Venlo, Netherlands). Approximately 30 mg of frozen swine bladder tissue was processed per sample. Tissue disruption and homogenization were performed with the gentleMACS Octo Dissociator (cat. no. 130‐134‐029) and M Tubes (cat. no. 130‐093‐236, Miltenyi Biotec, Bergisch Gladbach, Germany) using the manufacturer's pre‐programmed RNA‐02 protocol, in Buffer RLT Plus (cat. no. 1053393, Qiagen) supplemented with β‐mercaptoethanol (cat. no. 125470100, Thermo Fisher Scientific, Waltham, MA, USA). Homogenized lysates were centrifuged at 4000 × *g* for 3 min, and the supernatants were collected and transferred to gDNA Eliminator spin columns (included in cat. no. 74134, Qiagen) to remove genomic DNA. Subsequent RNA purification was performed according to the manufacturer's instructions, and RNA was eluted in 30 μL of TE Buffer. RNA yield and purity were assessed spectrophotometrically (EzDrop1000, Blue‐Ray Biotech, Xindian District, New Taipei City, Taiwan), with A260/A280 ratios >1.8 considered acceptable. The isolated RNAs were stored at −80°C until use.

### 2.5 RNA sequencing

cDNA libraries for next‐generation sequencing were prepared using the Zymo‐Seq RiboFree Total RNA Library Kit (cat. no. R3000, Zymo Research, Irvine, CA, USA). Briefly, total RNA was reverse transcribed using a mixture of oligo(dT) and random hexamer primers. The resulting cDNA was purified following the manufacturer's protocol, and PCR amplification was performed to both enrich and barcode the libraries, yielding Illumina‐compatible products. Library quality and concentration were assessed using a Bioanalyzer (Agilent Technologies, Santa Clara, CA, USA) and Qubit fluorometer (cat. no. Q33238, Thermo Fisher Scientific, Waltham, MA, USA). Final libraries were quantified by KAPA qPCR (Roche Sequencing, Pleasanton, CA, USA) prior to sequencing on the NovaSeq X Plus platform (Illumina, San Diego, CA, USA) at the UC Irvine Genomics Research and Technology Hub Core Facility.

### 2.6 In silico evaluations

Raw sequencing reads were trimmed and quality‐checked using CLC Genomics Workbench version 25.0.2 (Qiagen, Hilden, Germany), then aligned to the swine (*Sus scrofa*) reference genome (*Sscrofa*11.1). All sequencing data were normalized to TPM (Transcripts Per Million) prior to analysis. Differential gene expression analysis was subsequently performed within the same platform. Pathway enrichment analysis was conducted using Gene Set Enrichment Analysis (GSEA) within the Reactome database (V93, www.reactome.org). Genes with an absolute fold change >2 and a false discovery rate (FDR) <0.05 were considered significant and included in pathway enrichment analysis.

### 2.7 Real‐time reverse transcription‐quantitative polymerase chain reaction (RT‐qPCR)

Gene expression analysis was performed using total RNA isolated as described above. Primers for target genes were designed with NCBI Primer‐BLAST (https://www.ncbi.nlm.nih.gov/tools/primer‐blast/) and validated for specificity using BLAST (https://blast.ncbi.nlm.nih.gov/Blast.cgi). Oligonucleotides were chemically synthesized by Integrated DNA Technologies, Inc. (Coralville, IA, USA). Primer performance was tested by PCR followed by gel electrophoresis. Final primer sequences are provided in Table S1. Reverse transcription was performed with the ProtoScript® II First Strand cDNA Synthesis Kit (cat. no. E6560S, New England Biolabs, Ipswich, MA, USA) according to the manufacturer's protocol. Quantitative‐PCR was conducted using iTaq Universal SYBR Green Supermix (cat. no. 1725121, Bio‐Rad, Hercules, CA, USA) on a CFX Connect Real‐Time PCR Detection System (Bio‐Rad) with 96‐well plates. Grubbs' test (*α* = 0.05) was used to identify and exclude statistical outliers.

### 2.8 Statistical analyses

Urodynamic data at baseline and post‐pBOO were evaluated using the Mann–Whitney *U* test. Quantitative histomorphometric data were analyzed with the Kruskal–Wallis test in combination with the post hoc Dunn's test for pairwise evaluations. A *p* < 0.05 for all statistical evaluations was considered significant. Quantitative measurements are displayed as mean ± standard deviation (SD). RNA sequencing data were compiled in Microsoft Excel 365 (Microsoft Corp., Redmond, WA, USA). Statistical analyses and graph preparation were performed using GraphPad Prism 9 (GraphPad Software, San Diego, CA, USA). RNA‐seq data analysis and figure generation were conducted in CLC Genomics Workbench (Qiagen, Hilden, Germany). Data are presented as mean ± standard deviation, with *p* < 0.05 considered statistically significant. For RNA‐seq analyses, a false discovery rate (FDR) threshold of <0.05 was used to determine statistical significance.

## 3 RESULTS

**FIGURE 2:**
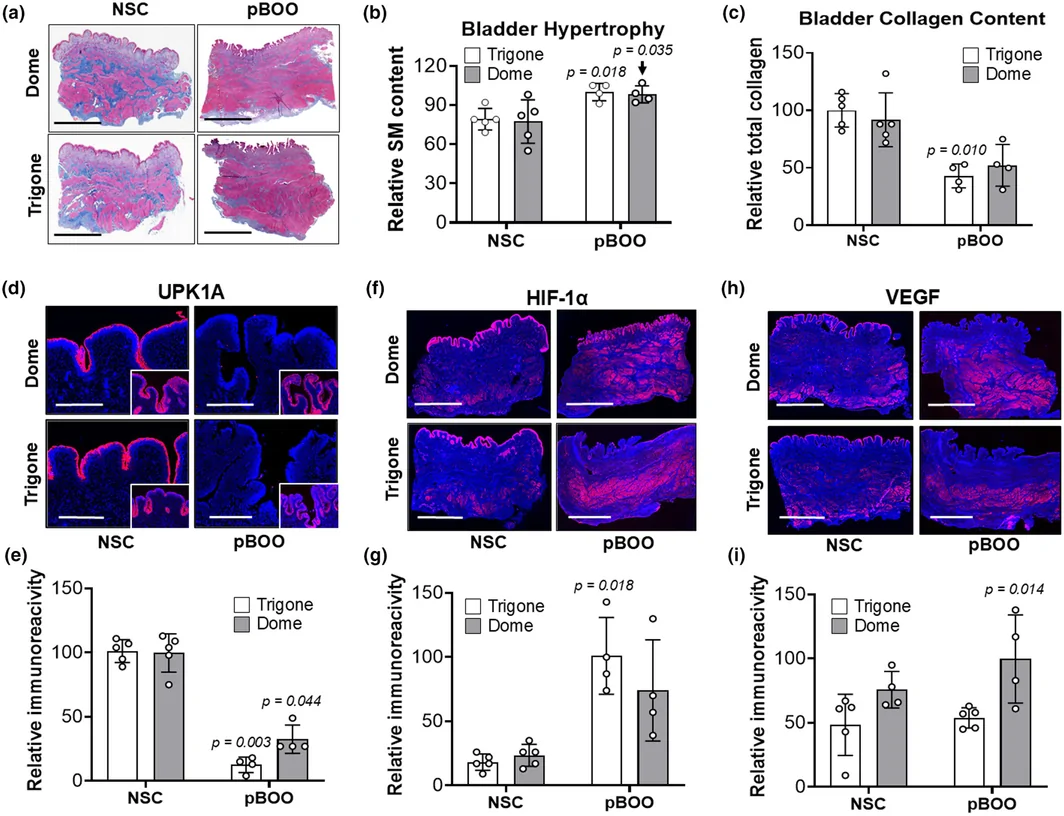
Histological, immunohistochemical, and histomorphometric analyses of the impact of acute pBOO on the porcine bladder dome and trigone. Representative photomicrographs of bladder dome and trigone cross‐sections in NSC and pBOO‐treated (4 weeks) swine subjected to Masson's trichrome staining (a) or immunoprobed for selective expression of UPK1A (d), pan‐CK (d, insets), HIF‐1ɑ (f), and VEGF (h). For panels d, f, and h, the respective marker expression is shown in red (Alexa Fluor 647 labeling) with DAPI nuclear counterstain displayed in blue. Scale bars for panels a, f and h are 5 mm and 200 μm for panel d. Histomorphometric quantitation of detrusor smooth muscle (b) and total collagen (c)content from MTS analysis as well as relative immunoreactivities of epithelial UPK1A (e) and detrusor HIF‐1ɑ (g) and VEGF (i) protein expression in bladder dome and trigone samples described above. *n* = 4–5 per group. Data in panels b, c, e, g, and i were analyzed using the Kruskal–Wallis test in combination with the post hoc Dunn's test and represented as means ± standard deviation. *p* values reflect comparisons to respective NSC values. CK, cytokeratin; HIF‐1ɑ, hypoxia inducible factor‐1α; pBOO, partial bladder outlet obstruction; UPK1A, uroplakin 1A.

All swine survived pBOO until scheduled euthanasia and displayed mild/moderate straining during voiding attempts. In addition, two pigs exhibited rectal prolapse during this period, which spontaneously recovered. No other intra‐operative or post‐operative complications were observed. Urodynamic evaluations of swine post‐pBOO demonstrated significant declines in bladder capacity and compliance, manifesting 35% ± 7% and 38% ± 10% of baseline values, respectively (Figure 1c). Cystographic assessments qualitatively confirmed reductions in bladder size in response to pBOO as detected by cystometry (Figure 1d). Histological and IHC analyses were performed on bladder dome and trigone regions in NSC and pBOO groups to characterize baseline and regional responses to obstructive uropathy. Significant elevations in smooth muscle content were detected in both the trigone and dome of porcine bladders following pBOO (Figure 2a,b), consistent with a hypertrophic phase of disease progression (Komninos & Mitsogiannis, 2014). In parallel, substantial declines in total collagen content were observed across both bladder regions in the pBOO group relative to NSC (Figure 2c). These findings mirror similar studies in rodent models of acute pBOO and are putatively the result of the coalescence of smooth muscle bundles in the hypertrophic detrusor and trigone during the early stages of pathologic bladder remodeling (Uvelius & Mattiasson, 1984). In addition, significant reductions in urothelial UPK1A immunoreactivity were observed across both anatomical locations relative to NSC counterparts (Figure 2d,e). On the other hand, hypoxic and angiogenic responses to pBOO were found to be regionally dependent, including selective upregulation of HIF‐1ɑ in the obstructed trigone (Figure 2f,g) as well as significant stimulation of VEGF protein expression in the pathological dome (Figure 2h,i). These region‐specific responses to pBOO may result from differential alterations in blood flow within the dome and trigone following bladder overdistension, since the dome is more susceptible to compression‐induced ischemia during filling (Sadahira et al., 2025; Schroder et al., 2001); however, this notion warrants further study. Overall, these results reveal that both global and region‐specific histopathological alterations occur in the porcine bladder following acute obstructive injury.

### 3.1 Global transcriptomic analysis of NSC versus obstructed bladder tissues

**FIGURE 3:**
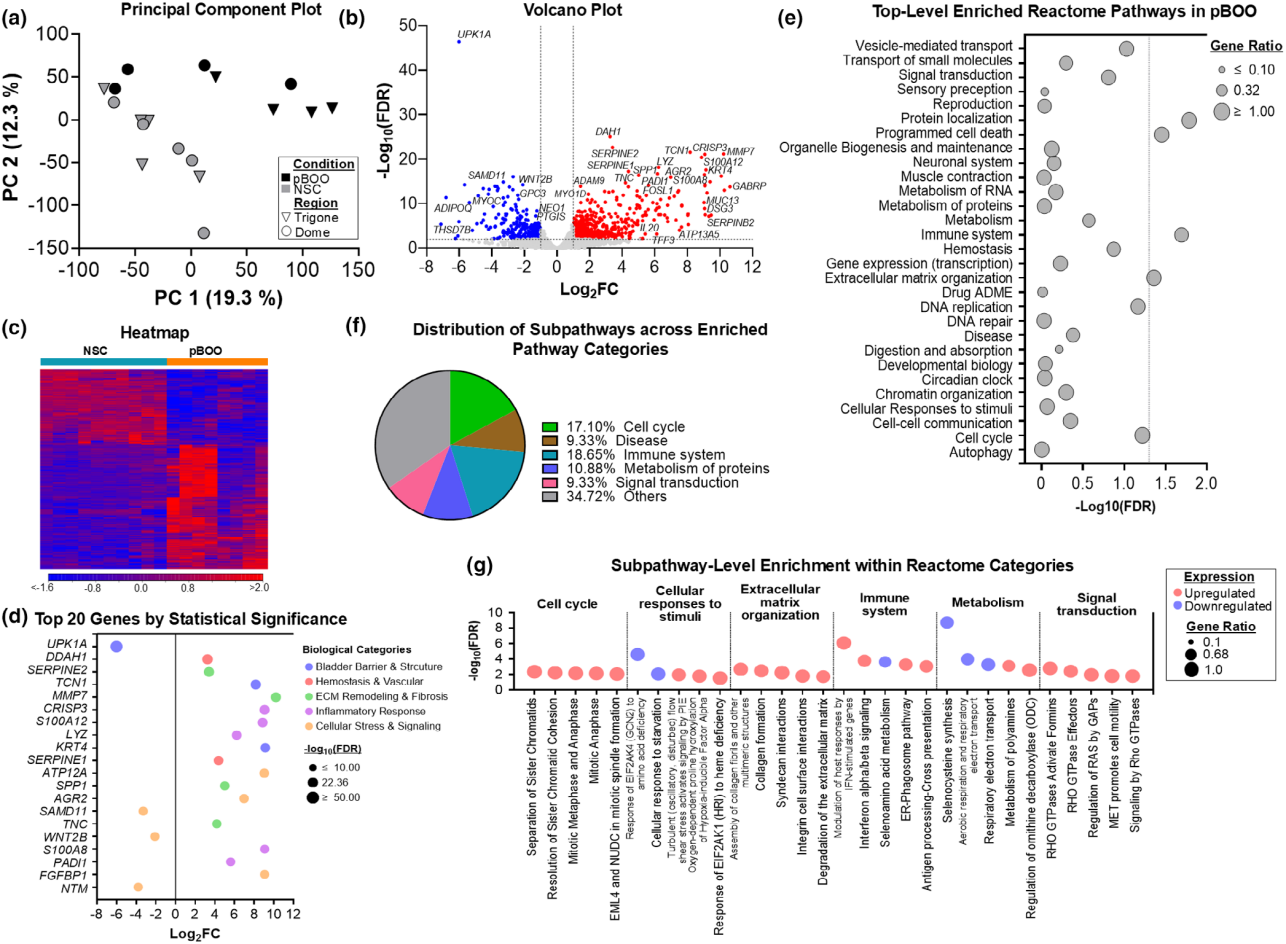
Transcriptomic profiling reveals distinct molecular signatures and pathway dysregulation in pBOO bladders. (a) Principal component analysis (PCA) of transcriptomic profiles from pBOO and NSC tissues. Orange indicates obstructed samples; cyan indicates normal tissues. Shapes denote anatomical regions (triangle = trigone, circle = dome). (b) Volcano plot displaying log_2_ fold change versus statistical significance (−log_10_(FDR)) for all genes. Red indicates significantly upregulated genes, blue indicates downregulated genes (FDR ≤0.01, |log_2_FC| ≥ 1). (c) Heatmap showing expression patterns of differentially expressed genes across NSC and pBOO samples. Color intensity reflects normalized expression levels. (d) Top 20 genes by statistical significance, categorized by biological function. Bubble size represents −log_10_(FDR). (e) Reactome pathway enrichment analysis showing −log_10_(FDR) values for top‐level pathways. Bubble size indicates gene ratio. (f) Distribution of enriched pathway categories (pie chart) with percentage representation of each functional group (FDR *p*‐value ≤0.05). (g) Subpathway‐level enrichment within major Reactome categories. Red bubbles indicate upregulated pathways; blue bubbles indicate downregulated pathways; bubble size represents gene ratio.

RNA sequencing analysis revealed widespread transcriptomic reprogramming in the bladder following pBOO. Principal component analysis demonstrated clear separation between obstructed and control samples (Figure 3a). Differential gene expression analysis identified 1998 differentially expressed genes (DEGs), including 491 significantly upregulated and 300 significantly downregulated genes (FDR ≤0.01, |log_2_ FC|≥1) in obstructed tissues compared with controls (Figure 3b). Hierarchical clustering further confirmed distinct expression profiles between the two groups (Figure 3c). Examination of the top 20 most significant genes (ranked by FDR) highlighted alterations in barrier function (*UPK1A*, *KRT4*), ECM remodeling (*MMP7*, *SERPINE2*, *SPP1*, *TNC*), inflammatory response (*S100A12*, *LYZ*, *CRISP3*), and hemostasis (*SERPINE1*), shown in Figure 3d. Notably, *UPK1A* was profoundly down‐regulated (log_2_FC = −8.22, FDR = 8.35 × 10^−19^), whereas *MMP7* was markedly up‐regulated (log_2_FC = 10.22, FDR = 6.51 × 10^−22^), indicating coordinated disruption of urothelial barrier integrity and enhanced tissue remodeling in obstructed bladders.

Pathway enrichment analysis revealed biological responses and functions following pBOO. Reactome Pathway analysis identified significant enrichment in ECM organization, immune system activation, and cell cycle pathways (Figure 3e). The distribution of enriched pathways showed a predominant representation of immune system processes (18.65%), cell cycle regulation (17.10%), and metabolism of proteins (10.88%) (Figure 3f). Subpathway‐level analysis further highlighted activation of ECM remodeling, inflammatory signaling, and cellular stress response pathways (Figure 3g). Full Reactome pathway enrichment is displayed in Table S2.

### 3.2 Regional differences in pBOO‐induced transcriptomic changes

**FIGURE 4:**
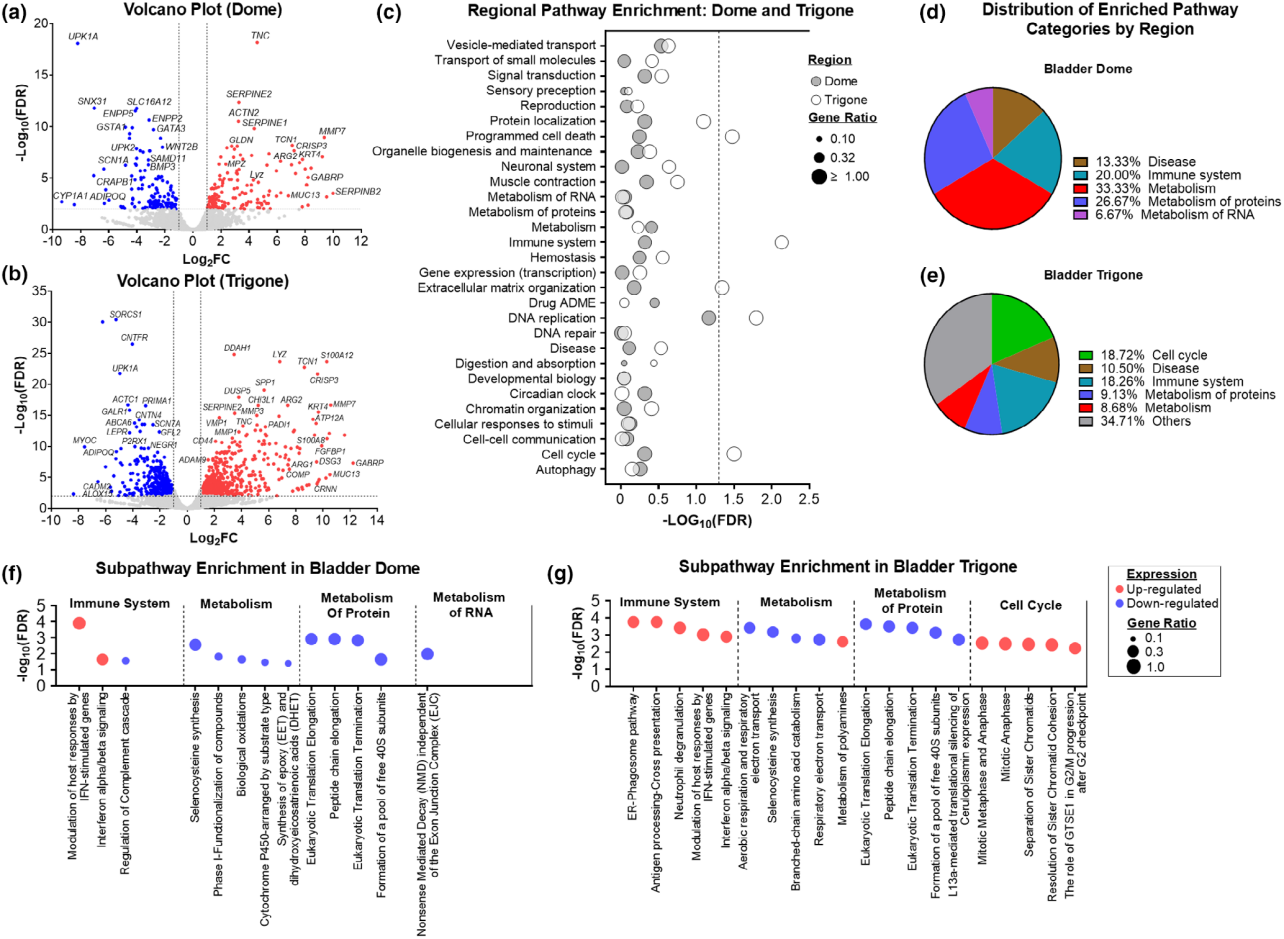
Regional transcriptome analysis reveals differential pathway activation between the bladder dome and the trigone following pBOO. (a, b) Volcano plots displaying log_2_ fold change versus statistical significance (−log_10_(FDR)) for bladder dome (a) and trigone (b) regions following pBOO. Red indicates significantly upregulated genes, blue indicates downregulated genes (FDR ≤0.01, |log_2_FC|≥1). (c) Regional comparison of Reactome pathway enrichment. Gray circles represent dome pathways; white circles represent trigone pathways. Bubble size indicates gene ratio. Vertical dashed line denotes the FDR = 0.05 threshold. (d, e) Distribution of enriched pathway categories by region. Pie charts show the percentage representation of major Reactome pathway categories in the dome (d) and the trigone (e). (f, g) Subpathway‐level enrichment within major Reactome categories for dome (f) and trigone (g). Red bubbles indicate upregulated pathways; blue indicates downregulated pathways. Bubble size represents gene ratio. Note the trigone‐specific enrichment of cell cycle pathways (g) and dome‐predominant metabolism pathways (f).

Although PCA analysis did not reveal a clear separation between the bladder dome and trigone (Figure 3a), differential expression analysis uncovered region‐specific transcriptomic alterations in response to pBOO. In the bladder dome tissue, 457 DEGs were identified, including 153 significantly upregulated and 144 significantly downregulated genes (FDR ≤0.01, |log_2_FC|≥1) compared with controls. In contrast, the trigone exhibited a markedly broader response, with 1769 DEGs, including 714 upregulated and 468 downregulated genes (FDR ≤0.01, |log_2_FC|≥1). Volcano plot analysis revealed both shared region‐specific transcriptomic responses to pBOO (Figure 4a,b). In both the dome and the trigone, *UPK1A* was significantly downregulated, indicating urothelial barrier disruption as a common feature of obstruction. Similarly, ECM remodeling genes (*MMP7*, *SERPINE2*) and inflammatory mediators (L*YZ*, *CRISP3*, *KRT4*) were upregulated in both regions, reflecting shared remodeling and inflammatory signatures. Despite these similarities, the trigone exhibited a substantially broader response, with approximately fourfold as many DEGs as the dome. Distinctive regional patterns were also observed. The dome showed greater downregulation of proliferative signaling (*WNT16, AXIN2*, and *DKK2*). Whereas the trigone uniquely showed downregulation of neurotrophic and metabolic regulators, including *SORCS1*, *CNTFR*, *MYOC*, *ADIPOQ*, and *MPZ*. Collectively, these results indicate that while dome and trigone share a core transcriptomic response involving barrier disruption, inflammation, and ECM remodeling, the trigone undergoes more extensive remodeling with additional suppression of neuronal and metabolic pathways.

A regional comparison of Reactome pathway enrichment revealed differential activation patterns between the dome and trigone (Figure 4c). The trigone showed significantly broader pathway activation across nearly all categories, with particularly strong enrichment in DNA replication, cell cycle regulation, and cellular responses to stimuli. These pathways were minimally activated or absent in the dome. Both regions exhibited activation of immune system pathways, though the trigone response was more extensive. The dome showed selective enrichment in metabolic pathways, including protein and RNA metabolism, whereas the trigone exhibited distinct activation of cell‐cycle and chromatin‐organization pathways.

Analysis of pathway category distribution revealed fundamental differences in molecular responses between regions (Figure 4d,e). The dome response was characterized by predominant activation of metabolism‐related pathways (33.33%), immune system responses (20.00%), and protein metabolism (26.67%). In contrast, the trigone exhibited a proliferative signature, with prominent activation of cell cycle pathways (18.72%) that was completely absent in the dome, alongside significant immune system activation (18.26%). A detailed examination of enriched subpathways revealed distinct functional responses across major pathway categories (Figure 4f,g). In the dome, immune system activation primarily involved interferon responses and complement cascades, while metabolic changes focused on selenoprotein synthesis, biological oxidation, and phase I drug metabolism. The trigone demonstrated more extensive subpathway activation across all categories, including robust immune responses such as ER‐phagosome interactions and antigen processing, comprehensive metabolic remodeling spanning amino acid metabolism and mitochondrial biogenesis, and prominent cell cycle regulation, including sister chromatid cohesion resolution and mitotic spindle checkpoint activation. Full Reactome pathway enrichment is shown in Tables S3 and S4.

These findings suggest that the bladder trigone serves as the primary molecular sensor and responder to pBOO, exhibiting extensive transcriptomic remodeling, with enrichment of proliferative and stress‐response pathways. In contrast, the dome shows a more limited response focused primarily on metabolic adaptation, further suggesting regional molecular responses following pBOO.

### 3.3 Core shared transcriptome reveals neuronal divergence between bladder dome and trigone responses to pBOO

**FIGURE 5:**
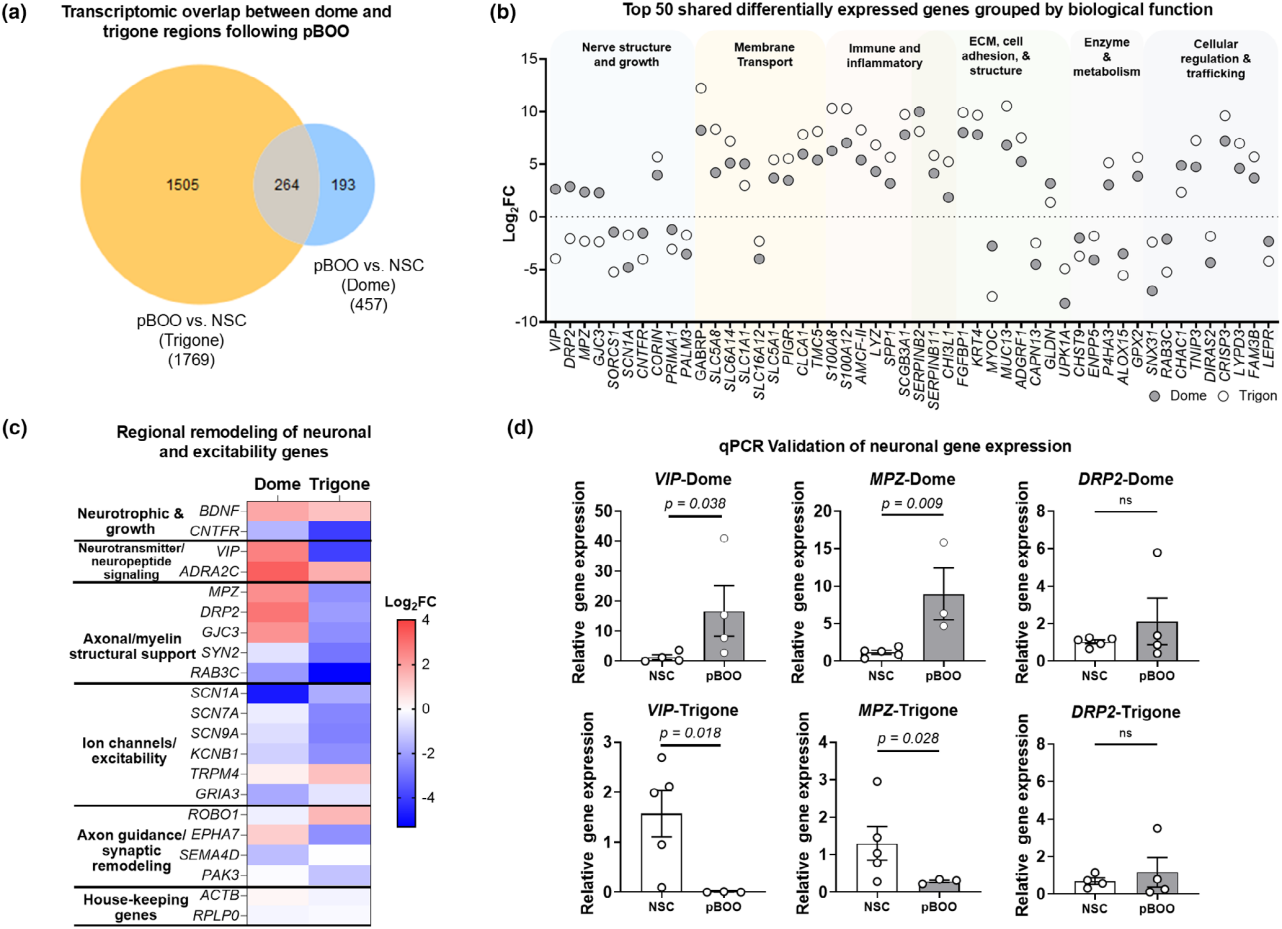
Shared and region‐specific transcriptomic responses in dome and trigone following pBOO. (a) Venn diagram showing the number of differentially expressed genes (DEGs) unique to the dome, the trigone, and overlapping between the regions. (b) Dot plot of the top 50 shared DEGs grouped by biological function, highlighting conserved signatures of extracellular matrix remodeling, immune activation, urothelial barrier disruption, and neuronal development. Several neuronal genes (*VIP, MPZ, DRP2, GJC3*) exhibited bidirectional expression, with upregulation in the dome and downregulation in the trigone. (c) Heatmap of curated neuro‐associated transcripts demonstrating both shared and region‐specific transcriptomic responses. *BDNF* was upregulated in both regions, whereas the dome exhibited upregulation of *VIP, MPZ, DRP2*, and *GJC3*, while the trigone showed suppression of these genes and additional downregulation of *CNTFR* and sodium channel genes (*SCN1A, SCN7A, SCN9A*). *ROBO1* was found to be upregulated in the trigone and downregulated in the dome. (d) qPCR confirmed the bidirectional regulation of *VIP* and *MPZ* between the dome and trigone, as identified by RNA sequencing. *DRP2* showed no significant differences between NSC and pBOO tissues. *ACTB* served as the housekeeping gene, and relative expression was calculated using the 2^−ΔΔCt^ method.

Pathway enrichment analysis revealed divergent functional responses in the dome and trigone following obstruction (Figure 4). To identify the core transcriptome associated with pBOO, DEGs that overlapped across regions were evaluated. This analysis revealed conserved sets of genes contributing to ECM remodeling, immune activation, urothelial barrier disruption, and neuronal development (Figure 5b). Several neuron‐associated genes (*VIP*, *MPZ*, *DRP2*, *GJC3*) showed bidirectional expression, upregulated in the dome but downregulated in the trigone, whereas other overlapping genes were mostly more strongly regulated (up‐ or downregulated) in the trigone than in the dome. The bidirectional expression of neuron‐associated genes prompted further evaluation of neuronal remodeling between the dome and trigone. Curated analysis of neuron‐associated transcripts revealed both shared and region‐specific responses to pBOO (Figure 5c). In both the dome and the trigone, *BDNF* was upregulated, indicating a common neuroplastic signature. Divergent patterns were also evident, as the dome exhibited upregulation of *VIP*, *MPZ*, *DRP2*, and *GJC3*, whereas the trigone showed strong downregulation of these same genes. Trigone‐specific suppression was further observed in *CNTFR* and multiple sodium channel genes (*SCN1A*, *SCN7A*, *SCN9A*), while *ROBO1* was uniquely upregulated. Collectively, these data suggest that while the dome and trigone share a subset of neurotrophic and signaling responses, the trigone undergoes broader suppression of neuronal structural and excitability‐related genes. *ACTB* and *RPLP0* were included as housekeeping controls for comparison.

Reverse transcription (RT)‐quantitative polymerase chain reaction (qPCR) was used to further validate the bidirectionally expressed neuron‐associated genes identified between the dome and the trigone in the RNA‐seq analysis. Figure 5d shows that *VIP* expression, encoding vasoactive intestinal peptide, was significantly higher in obstructed dome tissues (*p* = 0.038) and significantly lower in obstructed trigone tissues (*p* = 0.018), confirming the bidirectional regulation observed in the RNA‐seq data. Similarly, *MPZ*, a glycoprotein responsible for the structure of the peripheral myelin sheath, was significantly higher in obstructed dome tissues (*p* = 0.009), while markedly lower in the obstructed trigone tissues (*p* = 0.028), reflecting the same trend as the RNA‐seq data. In contrast, *DRP2*, another myelination‐associated gene important for peripheral nerve function, did not show significant differences between obstructed and control tissues in either region by qPCR.

## 4 DISCUSSION

Bladder remodeling secondary to anatomical or functional pBOO has traditionally been conceptualized as a progressive, organ‐wide process in which sustained bladder overdistension drives smooth muscle hypertrophy, culminating in fibrosis and functional decompensation (Fusco et al., 2018; Metcalfe et al., 2010). Existing pBOO models have largely characterized disease outcomes at the whole‐bladder level, implicitly treating the organ as transcriptionally and histologically homogeneous (Gheinani et al., 2017; Ito et al., 2021; Sidler et al., 2021; von Siebenthal et al., 2023). The aim of this report was to evaluate global and region‐specific transcriptomes in the porcine bladder following acute obstructive injury. Compared with NSC, pBOO‐treated swine exhibited low compliance, small‐capacity bladders with smooth muscle hypertrophy, global reductions in urothelial barrier protein expression, and regional upregulation of angiogenic and stress‐response proteins. These results are consistent with previously reported functional and histological alterations encountered in human and rodent studies of obstructive bladder diseases (Alsaikhan et al., 2015; Fusco et al., 2018; Komninos & Mitsogiannis, 2014; Zeber‐Lubecka et al., 2022), suggesting conserved pathophysiological processes exist between swine and other mammalian species.

In general, our findings show that the global transcriptome of obstructed porcine bladders mimicked molecular signatures encountered in published rodent (Duan et al., 2017; Ito et al., 2021; von Siebenthal et al., 2023; Zhu et al., 2022) and human (Akshay et al., 2024; Gheinani et al., 2018) evaluations of pBOO. Specifically, pathway‐level analysis of pBOO‐treated swine revealed enrichment of processes related to immune activation, cell cycle regulation, ECM organization, and signal transduction. Taken together, these pathways reflect the canonical sequence of inflammation, fibro‐proliferative remodeling, and aberrant ECM deposition described in rodent ligature models (Alsaikhan et al., 2015; Metcalfe et al., 2010), nerve‐sparing mid‐urethral obstruction (NeMO) model (Sidler et al., 2021), and human bladder obstruction transcriptomes (Akshay et al., 2024; Gheinani et al., 2017). Upregulation of ECM‐associated programs, including collagen biosynthesis and assembly, matrix metalloproteinase activity, and integrin‐mediated cell‐matrix interactions, closely parallels the ECM enrichment signature reported in the NeMO model (Sidler et al., 2021) and aligns with extensive evidence implicating altered collagen metabolism as a defining feature of pBOO‐induced pathologic remodeling (Fusco et al., 2018; Metcalfe et al., 2010). In parallel, subpathway‐level analysis identified consistent downregulation of aerobic respiration, mitochondrial electron transport, and respiratory chain components, mirroring suppression of oxidative phosphorylation and pyruvate metabolism reported in both rodent and human obstruction datasets (Akshay et al., 2024; Sidler et al., 2021). These findings are consistent with the energetic reprogramming that accompanies smooth muscle phenotypic modulation away from a contractile state (von Siebenthal et al., 2023). Collectively, this pathway‐level concordance supports the porcine model as a translational platform that captures conserved molecular features of pBOO.

This consistency extended to the transcript level, where profound downregulation of *UPK1A*, together with upregulation of *MMP7*, *S100A12*, *SPP1*, and *LYZ*, recapitulates gene‐level signatures reported across rodent and human obstruction datasets (Akshay et al., 2024; Gheinani et al., 2017), providing molecular validation beyond pathway enrichment alone. Specifically, previous rodent and human evaluations have shown that pBOO leads to hypoxic, ischemic, and direct cellular injury, which can cause urothelial dysfunction by disrupting the urine‐blood barrier via repression of cell junction proteins as well as UPK‐rich, asymmetric unit membranes (AUM) within the apical layer (Niemczyk et al., 2018). In particular, Romih and colleagues demonstrated that patients afflicted with pBOO displayed reductions in UPK expression and AUM formation compared to healthy individuals (Romih et al., 2003, 2008), while urothelial tight junctions and desmosomes were mitigated in rats with obstructive bladder damage (Celik‐Ozenci et al., 2006). These results are consistent with our current findings showing that porcine bladder tissues downregulate UPK (e.g., *UPK1A*) following pBOO, which may lead to alterations in urothelial barrier integrity. Concurrent with the loss of urothelial differentiation markers, the π subunit of the GABA(A) receptor (*GABRP*) was the most upregulated transcript in obstructed tissues (log_2_FC = 10.61, FDR = 1.68 × 10^−14^). Unlike classical neuronal GABA(A) subunits, GABRP is primarily expressed in non‐neuronal peripheral tissues and has been detected on smooth muscle cells of the urinary bladder, where GABAergic signaling has been proposed to regulate contractility (Hedblom & Kirkness, 1997; Quezada et al., 2006). Mechanistically, GABRP modulates cytoskeletal organization and cell motility via ERK1/2 signaling (Sizemore et al., 2014). Marked induction of GABRP expression in the obstructed bladder may therefore reflect a compensatory attempt to suppress pathological detrusor contractility, consistent with the broader urothelial signaling dysfunction and altered sensory transduction pathways reported in obstructed bladders (Fusco et al., 2018; Hedblom & Kirkness, 1997; Sadeghi & Taylor, 2010). Whether this represents an adaptive response or contributes to maladaptive remodeling remains to be determined.

Global activation of selective extracellular matrix remodeling genes in pBOO‐treated swine was also correlated to adaptive responses previously reported in human and rodent studies of obstructive uropathy (Aitken et al., 2010). For instance, mRNA expression of the matrix metalloproteinase gene, *MMP7*, was markedly upregulated in obstructed porcine bladders, which parallels results from Aitken et al., who demonstrated significant induction of this collagenase in rat bladders following pBOO as well as in bladder smooth muscle cells cultured in hypoxic conditions and under in vitro mechanical strain (Aitken et al., 2010). Elevated *MMP* expression during early stages of pBOO is hypothesized to play a key role in smooth muscle hypertrophy by degrading ECM components to allow for expansion of smooth muscle bundles within the bladder wall (Fusco et al., 2018) as well as facilitate the release of ECM‐sequestered mitogens such as epidermal growth factor and basic fibroblastic growth factor to promote smooth muscle cell growth (Chen et al., 1995). In addition, transcriptional targets of the pro‐fibrotic, transforming growth factor‐β (TGF‐β) signaling cascade, including *TNC*, *SPP1*, and *SERPINE1*, were also upregulated in pBOO‐treated swine, indicative of an ongoing stage of pathological detrusor tissue remodeling (Metcalfe et al., 2010). Similarly, enrichment of innate immune pathways is reflected in the upregulation of *S100A8*, *S100A12*, *LYZ*, and *CRISP3*, consistent with macrophage‐driven inflammatory activation previously implicated in obstructive bladder dysfunction across species (Sidler et al., 2021). Collectively, these transcriptional hallmarks, observed across rodent models (Metcalfe et al., 2010; Sidler et al., 2021; von Siebenthal et al., 2023) and human obstruction datasets (Akshay et al., 2024; Gheinani et al., 2017), further confirm that our porcine model captures the essential molecular pathology of pBOO.

While prior studies have largely attributed metabolic alterations during pBOO to hypoxia and a shift toward anaerobic glycolysis (Komninos & Mitsogiannis, 2014), our global transcriptomic analysis identified a striking downregulation of *ADIPOQ* in the obstructed bladder (log_2_FC = −5.38; FDR = 6.22E‐11). *ADIPOQ* encodes adiponectin, a key regulator of metabolic homeostasis that promotes fatty acid oxidation and glucose utilization by activating AMPK signaling and related metabolic pathways (Yamauchi et al., 2002). Recent studies of global ADIPOQ‐knockout mice have shown they exhibit lower urinary tract symptoms, including increased voiding frequency, small void volumes, and reduced bladder smooth muscle contractility due to disruptions in purinergic signaling (Luo et al., 2025). However, adiponectin signaling has received little attention in the context of pBOO. The global reduction in *ADIPOQ* within the pBOO‐treated group suggests that this pathology may disrupt pathways involved in cellular energy balance within the bladder, and therefore future investigations are warranted to determine the role of the adiponectin‐AMPK signaling axis in disease progression.

Despite global transcriptional concordance, regional analysis revealed that the molecular response to pBOO is highly regional rather than uniformly distributed across the bladder wall. The trigone exhibited nearly fourfold more differentially expressed genes than the dome (1769 vs. 457 DEGs; FDR ≤0.01, |log_2_FC|≥1). Beyond differences in response magnitude, these regions prioritized distinct biological processes. Reactome pathway analysis indicated that the obstructed trigone represents the epicenter of structural and inflammatory remodeling, with strong enrichment of cell cycle (18.72%) and immune system (18.26%) pathways. In contrast, the dome displayed a transcriptional profile dominated by metabolic processes, including general metabolism (33.33%) and protein metabolism (26.67%), with minimal enrichment of structural or inflammatory pathways.

In contrast to the highly proliferative and transcriptionally active profile observed in the trigone, the obstructed dome displayed enrichment patterns consistent with reduced biosynthetic activity. Downregulation of pathways involved in eukaryotic translation elongation and peptide chain elongation suggests a regional reduction in translational capacity, accompanied by suppression of WNT signaling components, including *AXIN2* and *WNT16*. In addition, the dome uniquely exhibited downregulation of cytochrome P450 and biological oxidation pathways, indicating that oxidative stress–related transcriptional programs may be spatially restricted rather than uniformly distributed across the bladder wall. Beyond the hypoxic and glycolytic adaptations frequently described in pBOO (Komninos & Mitsogiannis, 2014), the dome transcriptome also revealed suppression of lipid metabolism pathways, including downregulation of key regulators such as *PPARG* and *CIDEA*. Collectively, these findings suggest that regional metabolic remodeling contributes to anatomical heterogeneity in bladder adaptation to pBOO. Suppression of lipid metabolic regulators, including PPARG and CIDEA, in the dome may indicate reduced metabolic flexibility in this region, raising the possibility that dome tissue may be particularly vulnerable to energetic stress during obstruction. In contrast, the proliferative transcriptional profile observed in the trigone may reflect continued metabolic activity that temporarily preserves contractile function despite ongoing fibrotic remodeling. These observations raise the hypothesis that metabolic vulnerability of the dome represents an early feature of pBOO‐induced bladder remodeling that could precede overt decompensation.

Within this spatially heterogeneous response, neuronal signaling pathways emerged as another regionally patterned feature of pBOO‐induced bladder remodeling. The present dataset revealed a neuronal remodeling signature with both shared and spatially divergent components across bladder regions. Both the dome and trigone exhibited a common neurotrophic response that may represent an early adaptive mechanism to maintain innervation during pBOO. Brain‐derived neurotrophic factor (*BDNF*) was significantly upregulated across obstructed tissues, consistent with neuroplastic responses reported during compensatory hypertrophy in pBOO models (Gao et al., 2021). Despite this shared neurotrophic activation, multiple genes associated with neuronal structure and excitability displayed striking zone‐specific divergence. The bidirectional expression of *VIP*, which was upregulated in the dome yet suppressed in the trigone, is particularly notable given that reduced density of VIP‐immunoreactive nerve fibers has been reported in obstructed bladders (Chapple et al., 1992). The present data suggest that this loss of *VIP* signaling may not be uniform across the bladder wall but may instead be concentrated in the trigone, whereas the dome maintains or augments *VIP* expression. The opposing regulation of *VIP* in the dome and trigone, despite shared *BDNF* upregulation, suggests region‐specific regulatory influences that may modulate responses to neurotrophic signaling, potentially reflecting differences in local inflammatory or hypoxic environments. A similar bidirectional pattern was observed for the *MPZ*, *DRP2*, and *GJC3*, each of which was upregulated in the dome yet suppressed in the trigone. Bidirectional regulation of both *VIP* (*p* = 0.038 dome, *p* = 0.018 trigone) and *MPZ* (*p* = 0.009 dome, *p* = 0.028 trigone) was confirmed by qPCR, whereas *DRP2* did not show significant differences by qPCR despite the directional changes observed in the transcriptomic dataset. The trigone also displayed selective downregulation of the ciliary neurotrophic factor receptor (*CNTFR*) and several voltage‐gated sodium channel transcripts (*SCN1A*, *SCN7A*, *SCN9A*), whereas the *ROBO1* transcript was uniquely upregulated in this region. Collectively, these findings suggest that although both regions initiate a common neurotrophic response, the trigone exhibits a broader suppression of genes that support axonal structure and neuronal excitability, consistent with region‐specific neuronal vulnerability. Clinical studies have documented progressive loss of autonomic innervation in obstructed human bladders, including reduced cholinergic nerve density and depletion of sensory neuropeptide‐immunoreactive fibers (Chapple et al., 1992; Gosling et al., 1986), yet the spatial distribution of this denervation within the bladder wall has not previously been characterized at the transcriptomic level. Together, these observations suggest that neuronal vulnerability during obstruction may be spatially heterogeneous, with preferential involvement of the trigone.

The pronounced anatomical heterogeneity identified in this study carries important implications for experimental sampling and therapeutic strategy. Tissue‐based molecular studies of pBOO frequently rely on samples obtained from a single anatomical region without accounting for spatial variation across the bladder wall. As demonstrated here, such sampling approaches may underestimate the severity of remodeling if tissue is obtained from the dome rather than the trigone. Similarly, candidate biomarkers derived from whole‐bladder homogenates or single‐region analyses may fail to capture the full dynamic range of obstruction‐driven transcriptional changes. The regional concentration of fibrotic remodeling within the trigone and metabolic alterations within the dome suggest that anatomical context may be important when interpreting molecular signatures of disease progression. From a therapeutic perspective, the zone‐specific divergence observed across hypoxic, fibrotic, metabolic, and neuronal pathways suggests that interventions targeting pBOO‐induced pathology should account for regional heterogeneity in target expression, rather than assuming uniform remodeling across the bladder wall. Furthermore, this regional diversity in pathologic responses creates an opportunity to explore focal intravesical treatments to mitigate pBOO‐induced detrusor dysfunction.

Several limitations of the present study should be acknowledged. The sample size, while consistent with large‐animal transcriptomic studies, limits statistical power for detecting subtle regional differences and precludes robust subgroup analyses. Although the dataset clearly identified major transcriptional shifts between pBOO and NSC tissues, some biologically relevant regional interactions may remain underpowered. This study also captures a single time point under selective pBOO conditions. Consequently, the temporal sequence of spatial divergence cannot be resolved, and it remains unclear whether the trigone‐dominant remodeling pattern is established early after obstruction or emerges progressively over the course of the disease (Akshay et al., 2024). The use of bulk RNA sequencing further prevents attribution of observed gene expression changes to specific cell populations. As a result, some regional differences may reflect variation in cellular composition rather than cell‐autonomous transcriptional reprogramming. Future studies incorporating single‐cell and spatial transcriptomic approaches will therefore be required to define the cellular basis of the trigone‐dominant remodeling gradient. Finally, although the porcine model offers important anatomical and physiological advantages over rodent systems, translation of these findings to human bladder obstruction will require validation in clinical specimens with matched regional sampling. Longitudinal studies integrating temporal sampling with single‐cell and spatial transcriptomics will be essential to determine whether early regional transcriptional signatures predict the transition from compensatory remodeling to bladder decompensation.

## 5 CONCLUSIONS

This study established comprehensive global and region‐specific transcriptomic atlases of the porcine bladder in response to pBOO through RNA sequencing. Our results show that the global transcriptome of the obstructed porcine bladder recapitulates conserved molecular signatures observed in human and rodent evaluations of pBOO, including enrichment of processes related to loss of urothelial barrier function, immune activation, cell cycle regulation, ECM organization, and signal transduction. Most importantly, we also uncovered that adaptive responses to pBOO are spatially encoded rather than globally uniform, with region‐specific divergence observed across hypoxic, fibrotic, metabolic, and neuronal pathways between the obstructed dome and trigone. These findings highlight the regional heterogeneity of bladder responses to obstructive uropathy and may inform future therapeutic interventions based on selective zonal drug targeting.

## AUTHOR CONTRIBUTIONS

**Yi Xi Wu:** Conceptualization; data curation; formal analysis; funding acquisition; investigation; methodology; project administration; supervision; visualization. **Gokhan Gundogdu:** Conceptualization; data curation; formal analysis; funding acquisition; investigation; methodology. **Madison Rivero:** Data curation; formal analysis; funding acquisition; investigation; methodology. **Caila Ruiz‐May:** Data curation; formal analysis; funding acquisition; investigation; methodology. **Ahmet Tarik Gundogdu:** Data curation; formal analysis; funding acquisition; investigation; methodology. **Victor Pham:** Data curation; formal analysis; funding acquisition; investigation; methodology. **Keith Rourke:** Data curation; formal analysis; funding acquisition; investigation. **Zhina Sadeghi:** Data curation; formal analysis; funding acquisition; investigation. **Joel Gelman:** Data curation; formal analysis; funding acquisition; investigation. **Joshua R. Mauney:** Conceptualization; data curation; formal analysis; funding acquisition; investigation; methodology; project administration; supervision; visualization.

## FUNDING INFORMATION

This study was supported by the National Institutes of Health Grants, R01DK131211 (Mauney), R01DK119240 (Mauney), R21AG093287 (Wu and Sadeghi), K08DK142008 (Sadeghi), as well as generous support from the Randy Douthit and Jerry D. Choate Tissue Engineering Fund (Gelman). The funding sources had no role in study design; in the collection, analysis, and interpretation of data; in the writing of the report; or in the decision to submit the article for publication.

## CONFLICT OF INTEREST STATEMENT

None declared.

## ETHICS STATEMENT

All animal experiments were approved by the Institutional Animal Care and Use Committee of the University of California, Irvine under protocol AUP‐22‐130.

## Supporting information


**Table S1.** Quantitative PCR (qPCR) Primer sequences.
**Table S2.** Full Reactome enriched pathways of global transcriptomic analysis of pBOO versus NSC bladder tissue. Pathways are filtered by *p*‐values ≤0.005; displayed pathways are based on FDR *p*‐values ≤0.05.
**Table S3.** Full Reactome enriched pathways of trigone transcriptomic analysis of pBOO versus NSC bladder tissue. Pathways are filtered by *p*‐values ≤0.005; displayed pathways are based on FDR *p*‐values ≤0.05.
**Table S4.** Full Reactome enriched pathways of dome transcriptomic analysis of obstructed versus NSC bladder tissue. Pathways are filtered by *p*‐values ≤0.005; displayed pathways are based on FDR *p*‐values ≤0.05.

## Data Availability

The transcriptomic sequencing datasets generated and analyzed during this study have been deposited in the NCBI Gene Expression Omnibus (GEO) under accession number GSE337305. This data is publicly available through the GEO repository. Supplemental data are deposited in FigShare and are publicly available.
