# Supplementary material for: Characterization of global and region‐specific transcriptomic alterations in the bladder following acute partial bladder outlet obstruction in female mini‐swine
Source: Physiol Rep. 2026 Aug 19;14(16):e71074. doi: 10.14814/phy2.71074 (PMC13490926; doi:10.14814/phy2.71074)
Supplement: Supplementary file 1 [file PHY2-14-e71074-s001.docx]

**Supplemental Information**

**Table S1. Quantitative PCR (qPCR) Primer sequences.**

| **Gene** | **Primer ID** |  | **Sequence (5’ -> 3’)** | **Amplicon Length** |
| --- | --- | --- | --- | --- |
| VIP | MW 415 | F | GCGGAGCATTGAAGGAGAA | 107 bps |
|  | MW 416 | R | CCTTCAAGAATTCACTGGGTAGT |  |
| MPZ | MW 323 | F | TCTCCTCTTTGGTGCTCTCC | 127 bps |
|  | MW 324 | R | TCATCCGAGACCCACTCACT |  |
| DRP2 | MW 335 | F | CACCTTGGTGTCCTGCTTCA | 124 bps |
|  | MW 336 | R | GCTTCCCAGTGCTAAAACGG |  |
| Beta-Actin | MW 55 | F | GTTCTGGCTCTTCTGCTCTC | 125 bps |
|  | MW 56 | R | ACAGTAGGCACTCAGCTACTA |  |

**Table S2. Full Reactome enriched pathways of global transcriptomic analysis of pBOO vs. NSC bladder tissue.** Pathways are filtered by p-values ≤ 0.005; displayed pathways are based on FDR p-values ≤ 0.05.

| **Categories** | **Pathway name** | **Entities found** | **Entities Total** | **Entities p-Value** | **Entities FDR** | **Expression** |
| --- | --- | --- | --- | --- | --- | --- |
| **Autophagy** | Pexophagy | 11 | 13 | 2.99E-03 | 4.38E-02 | ▼▼ |
| **Cell cycle** | Separation of Sister Chromatids | 159 | 182 | 6.40E-05 | 4.44E-03 | ▲▲ |
|  | Resolution of Sister Chromatid Cohesion | 114 | 135 | 1.11E-04 | 6.07E-03 | ▲▲ |
|  | Mitotic Metaphase and Anaphase | 205 | 237 | 1.36E-04 | 7.02E-03 | ▲▲ |
|  | Mitotic Anaphase | 204 | 236 | 1.45E-04 | 7.35E-03 | ▲▲ |
|  | EML4 and NUDC in mitotic spindle formation | 104 | 121 | 1.97E-04 | 8.97E-03 | ▲▲ |
|  | Amplification of signal from unattached kinetochores via a MAD2 inhibitory signal | 89 | 94 | 2.80E-04 | 1.13E-02 | ▲▲ |
|  | Amplification of signal from the kinetochores | 89 | 94 | 2.80E-04 | 1.13E-02 | ▲▲ |
|  | Condensation of Prometaphase Chromosomes | 10 | 15 | 2.85E-04 | 1.13E-02 | ▲▲ |
|  | The role of GTSE1 in G2/M progression after G2 checkpoint | 53 | 70 | 3.53E-04 | 1.31E-02 | ▲▲ |
|  | Activation of APC/C and APC/C:Cdc20 mediated degradation of mitotic proteins | 57 | 64 | 5.77E-04 | 1.75E-02 | ▲▲ |
|  | Cell Cycle Checkpoints | 232 | 266 | 7.45E-04 | 1.89E-02 | ▲▲ |
|  | APC/C:Cdc20 mediated degradation of mitotic proteins | 56 | 63 | 8.18E-04 | 1.95E-02 | ▲▲ |
|  | Orc1 removal from chromatin | 52 | 60 | 8.71E-04 | 2.00E-02 | ▲▲ |
|  | M Phase | 325 | 403 | 9.18E-04 | 2.03E-02 | ▲▲ |
|  | Cell Cycle, Mitotic | 482 | 586 | 9.20E-04 | 2.03E-02 | ▲▲ |
|  | Regulation of mitotic cell cycle | 67 | 79 | 9.54E-04 | 2.03E-02 | ▲▲ |
|  | APC/C-mediated degradation of cell cycle proteins | 67 | 79 | 9.54E-04 | 2.03E-02 | ▲▲ |
|  | Mitotic G1 phase and G1/S transition | 145 | 163 | 9.81E-04 | 2.05E-02 | ▲▲ |
|  | Regulation of APC/C activators between G1/S and early anaphase | 62 | 70 | 1.06E-03 | 2.18E-02 | ▲▲ |
|  | Mitotic Spindle Checkpoint | 105 | 111 | 1.10E-03 | 2.22E-02 | ▲▲ |
|  | p53-Independent DNA Damage Response | 34 | 41 | 1.11E-03 | 2.22E-02 | ▲▲ |
|  | Ubiquitin Mediated Degradation of Phosphorylated Cdc25A | 34 | 41 | 1.11E-03 | 2.22E-02 | ▲▲ |
|  | p53-Independent G1/S DNA damage checkpoint | 34 | 41 | 1.11E-03 | 2.22E-02 | ▲▲ |
|  | Mitotic Prometaphase | 176 | 212 | 1.15E-03 | 2.25E-02 | ▲▲ |
|  | APC:Cdc20 mediated degradation of cell cycle proteins prior to satisfation of the cell cycle checkpoint | 55 | 61 | 1.19E-03 | 2.25E-02 | ▲▲ |
|  | Synthesis of DNA | 101 | 119 | 1.35E-03 | 2.46E-02 | ▲▲ |
|  | Cdc20:Phospho-APC/C mediated degradation of Cyclin A | 54 | 60 | 1.86E-03 | 3.17E-02 | ▲▲ |
|  | SCF-beta-TrCP mediated degradation of Emi1 | 38 | 42 | 1.91E-03 | 3.22E-02 | ▲▲ |
|  | CDK-mediated phosphorylation and removal of Cdc6 | 54 | 62 | 2.26E-03 | 3.68E-02 | ▲▲ |
|  | Switching of origins to a post-replicative state | 72 | 81 | 2.38E-03 | 3.83E-02 | ▲▲ |
|  | Ubiquitin-dependent degradation of Cyclin D | 35 | 41 | 2.96E-03 | 4.38E-02 | ▲▲ |
|  | SCF(Skp2)-mediated degradation of p27/p21 | 45 | 52 | 3.08E-03 | 4.47E-02 | ▲▲ |
|  | G1/S Transition | 122 | 140 | 3.26E-03 | 4.62E-02 | ▲▲ |
| **Cell-cell communication** | Type I hemidesmosome assembly | 10 | 11 | 9.32E-04 | 2.03E-02 | ▲▲ |
| **Cellular responses to stimuli** | Response of EIF2AK4 (GCN2) to amino acid deficiency | 86 | 111 | 1.28E-07 | 2.60E-05 | ▼▼ |
|  | Cellular response to starvation | 135 | 172 | 1.83E-04 | 8.79E-03 | ▼▼ |
|  | Turbulent (oscillatory, disturbed) flow shear stress activates signaling by PIEZO1 and integrins in endothelial cells | 31 | 42 | 2.88E-04 | 1.13E-02 | ▲▲ |
|  | Oxygen-dependent proline hydroxylation of Hypoxia-inducible Factor Alpha | 47 | 59 | 5.13E-04 | 1.65E-02 | ▲▲ |
|  | Response of EIF2AK1 (HRI) to heme deficiency | 25 | 29 | 1.70E-03 | 2.93E-02 | ▲▲ |
|  | Unfolded Protein Response (UPR) | 146 | 156 | 2.52E-03 | 3.98E-02 | ▲▲ |
|  | Cellular response to hypoxia | 59 | 73 | 2.94E-03 | 4.38E-02 | ▲▲ |
| **Developmental biology** | Somitogenesis | 40 | 53 | 4.28E-05 | 3.36E-03 | ▲▲ |
|  | EPHB-mediated forward signaling | 40 | 52 | 5.38E-05 | 3.94E-03 | ▲▲ |
|  | EPH-Ephrin signaling | 85 | 102 | 9.26E-04 | 2.03E-02 | ▲▲ |
|  | Formation of paraxial mesoderm | 52 | 69 | 2.94E-03 | 4.38E-02 | ▲▲ |
| **Disease** | Viral mRNA Translation | 68 | 101 | 3.56E-12 | 1.91E-09 | ▼▼ |
|  | SARS-CoV-1 modulates host translation machinery | 27 | 40 | 6.44E-09 | 1.89E-06 | ▼▼ |
|  | Influenza Viral RNA Transcription and Replication | 115 | 154 | 1.07E-06 | 1.57E-04 | ▼▼ |
|  | SARS-CoV-2 modulates host translation machinery | 40 | 56 | 2.53E-05 | 2.38E-03 | ▼▼ |
|  | FCGR3A-mediated phagocytosis | 52 | 157 | 7.20E-05 | 4.52E-03 | ▲▲ |
|  | Parasite infection | 52 | 157 | 7.20E-05 | 4.52E-03 | ▲▲ |
|  | Leishmania phagocytosis | 52 | 157 | 7.20E-05 | 4.52E-03 | ▲▲ |
|  | Hh mutants are degraded by ERAD | 39 | 48 | 5.35E-04 | 1.68E-02 | ▲▲ |
|  | Influenza Infection | 133 | 179 | 6.11E-04 | 1.75E-02 | ▼▼ |
|  | CD163 mediating an anti-inflammatory response | 9 | 14 | 7.22E-04 | 1.89E-02 | ▲▲ |
|  | Hh mutants abrogate ligand secretion | 42 | 51 | 7.27E-04 | 1.89E-02 | ▲▲ |
|  | Maturation of spike protein | 37 | 48 | 9.25E-04 | 2.03E-02 | ▲▲ |
|  | Late SARS-CoV-2 Infection Events | 64 | 102 | 1.17E-03 | 2.25E-02 | ▲▲ |
|  | Translation of Structural Proteins | 54 | 85 | 1.70E-03 | 2.93E-02 | ▲▲ |
|  | Diseases of branched-chain amino acid catabolism | 13 | 32 | 2.44E-03 | 3.90E-02 | ▼▼ |
|  | Microbial modulation of RIPK1-mediated regulated necrosis | 4 | 10 | 2.83E-03 | 4.36E-02 | ▲▲ |
|  | Vif-mediated degradation of APOBEC3G | 36 | 43 | 3.10E-03 | 4.47E-02 | ▲▲ |
|  | FLT3 signaling by CBL mutants | 7 | 7 | 3.54E-03 | 4.83E-02 | ▼▼ |
| **DNA Replication** | Assembly of the pre-replicative complex | 80 | 103 | 3.20E-03 | 4.58E-02 | ▲▲ |
|  | DNA Replication Pre-Initiation | 96 | 121 | 3.35E-03 | 4.69E-02 | ▲▲ |
| **Drug ADME** | Ribavirin ADME | 10 | 27 | 2.85E-03 | 4.36E-02 | ▲▲ |
| **Extracellular matrix organization** | Assembly of collagen fibrils and other multimeric structures | 54 | 67 | 2.13E-05 | 2.08E-03 | ▲▲ |
|  | Collagen formation | 80 | 104 | 4.33E-05 | 3.36E-03 | ▲▲ |
|  | Syndecan interactions | 26 | 29 | 1.06E-04 | 5.93E-03 | ▲▲ |
|  | Integrin cell surface interactions | 70 | 86 | 4.97E-04 | 1.64E-02 | ▲▲ |
|  | Degradation of the extracellular matrix | 108 | 148 | 7.72E-04 | 1.89E-02 | ▲▲ |
|  | Collagen degradation | 52 | 69 | 1.04E-03 | 2.15E-02 | ▲▲ |
|  | Extracellular matrix organization | 267 | 351 | 2.90E-03 | 4.38E-02 | ▲▲ |
| **Gene expression (Transcription)** | Regulation of RUNX3 expression and activity | 40 | 47 | 1.19E-03 | 2.25E-02 | ▲▲ |
| **Hemostasis** | Dissolution of Fibrin Clot | 9 | 14 | 2.05E-05 | 2.08E-03 | ▲▲ |
|  | Kinesins | 49 | 68 | 9.68E-04 | 2.04E-02 | ▲▲ |
|  | Platelet Adhesion to exposed collagen | 14 | 17 | 2.52E-03 | 3.98E-02 | ▲▲ |
|  | GPVI-mediated activation cascade | 31 | 43 | 3.40E-03 | 4.71E-02 | ▲▲ |
| **Immune system** | Modulation of host responses by IFN-stimulated genes | 18 | 22 | 2.51E-09 | 8.28E-07 | ▲▲ |
|  | Interferon alpha/beta signaling | 85 | 129 | 1.23E-06 | 1.70E-04 | ▲▲ |
|  | Selenoamino acid metabolism | 93 | 178 | 1.80E-06 | 2.38E-04 | ▼▼ |
|  | ER-Phagosome pathway | 66 | 98 | 4.17E-06 | 5.23E-04 | ▲▲ |
|  | Antigen processing-Cross presentation | 80 | 120 | 8.12E-06 | 8.92E-04 | ▲▲ |
|  | Cross-presentation of soluble exogenous antigens (endosomes) | 37 | 46 | 3.61E-05 | 3.17E-03 | ▲▲ |
|  | Signaling by Interleukins | 503 | 646 | 4.00E-05 | 3.36E-03 | ▲▲ |
|  | Cytokine Signaling in Immune system | 818 | 1,095 | 4.17E-05 | 3.36E-03 | ▲▲ |
|  | Regulation of actin dynamics for phagocytic cup formation | 52 | 158 | 7.00E-05 | 4.52E-03 | ▲▲ |
|  | Neutrophil degranulation | 365 | 478 | 1.54E-04 | 7.64E-03 | ▲▲ |
|  | MHC class II antigen presentation | 105 | 137 | 1.74E-04 | 8.51E-03 | ▲▲ |
|  | Interferon Signaling | 281 | 404 | 1.93E-04 | 8.92E-03 | ▲▲ |
|  | Interleukin-12 family signaling | 66 | 96 | 2.55E-04 | 1.10E-02 | ▲▲ |
|  | Interleukin-1 family signaling | 130 | 170 | 2.67E-04 | 1.12E-02 | ▲▲ |
|  | Innate Immune System | 784 | 1,334 | 3.11E-04 | 1.17E-02 | ▲▲ |
|  | Antigen processing: Ub, ATP-independent proteasomal degradation | 17 | 21 | 4.27E-04 | 1.56E-02 | ▲▲ |
|  | Antigen Presentation: Folding, assembly and peptide loading of class I MHC | 22 | 41 | 4.40E-04 | 1.59E-02 | ▲▲ |
|  | Downstream signaling events of B Cell Receptor (BCR) | 64 | 78 | 4.68E-04 | 1.59E-02 | ▲▲ |
|  | Fcgamma receptor (FCGR) dependent phagocytosis | 77 | 193 | 5.37E-04 | 1.68E-02 | ▲▲ |
|  | Activation of NF-kappaB in B cells | 50 | 59 | 6.05E-04 | 1.75E-02 | ▲▲ |
|  | NF-kB activation through FADD/RIP-1 pathway mediated by caspase-8 and -10 | 12 | 14 | 6.31E-04 | 1.77E-02 | ▲▲ |
|  | Dectin-1 mediated noncanonical NF-kB signaling | 45 | 53 | 7.05E-04 | 1.89E-02 | ▲▲ |
|  | TCR signaling | 91 | 122 | 7.47E-04 | 1.89E-02 | ▲▲ |
|  | NIK-->noncanonical NF-kB signaling | 42 | 48 | 7.54E-04 | 1.89E-02 | ▲▲ |
|  | Interleukin-4 and Interleukin-13 signaling | 164 | 211 | 7.55E-04 | 1.89E-02 | ▲▲ |
|  | Interleukin-1 signaling | 89 | 112 | 7.78E-04 | 1.89E-02 | ▲▲ |
|  | Immune System | 1770 | 2,664 | 9.03E-04 | 2.03E-02 | ▲▲ |
|  | C-type lectin receptors (CLRs) | 102 | 190 | 1.19E-03 | 2.25E-02 | ▲▲ |
|  | CLEC7A (Dectin-1) signaling | 80 | 107 | 1.45E-03 | 2.64E-02 | ▲▲ |
|  | PKR-mediated signaling | 61 | 88 | 1.48E-03 | 2.67E-02 | ▲▲ |
|  | Signaling by the B Cell Receptor (BCR) | 91 | 176 | 1.69E-03 | 2.93E-02 | ▲▲ |
|  | Toll-like Receptor Cascades | 156 | 210 | 2.62E-03 | 4.11E-02 | ▲▲ |
|  | Interferon gamma signaling | 109 | 177 | 3.07E-03 | 4.47E-02 | ▲▲ |
|  | Interleukin-27 signaling | 9 | 13 | 3.23E-03 | 4.61E-02 | ▲▲ |
|  | Interleukin-10 signaling | 62 | 86 | 3.31E-03 | 4.67E-02 | ▲▲ |
|  | Fc epsilon receptor (FCERI) signaling | 113 | 222 | 3.44E-03 | 4.75E-02 | ▲▲ |
| **Metabolism** | Selenocysteine synthesis | 71 | 108 | 3.63E-12 | 1.91E-09 | ▼▼ |
|  | Aerobic respiration and respiratory electron transport | 231 | 361 | 6.04E-07 | 1.06E-04 | ▼▼ |
|  | Respiratory electron transport | 142 | 197 | 4.51E-06 | 5.40E-04 | ▼▼ |
|  | Metabolism of polyamines | 39 | 67 | 6.81E-06 | 7.81E-04 | ▲▲ |
|  | Regulation of ornithine decarboxylase (ODC) | 33 | 38 | 3.00E-05 | 2.73E-03 | ▲▲ |
|  | Branched-chain amino acid catabolism | 21 | 59 | 5.90E-05 | 4.20E-03 | ▼▼ |
|  | Citric acid cycle (TCA cycle) | 34 | 83 | 4.61E-04 | 1.59E-02 | ▼▼ |
|  | Complex I biogenesis | 60 | 71 | 8.26E-04 | 1.95E-02 | ▼▼ |
|  | Maturation of TCA enzymes and regulation of TCA cycle | 19 | 47 | 1.65E-03 | 2.93E-02 | ▼▼ |
|  | Sulfur amino acid metabolism | 28 | 95 | 1.75E-03 | 3.00E-02 | ▼▼ |
|  | Complex IV assembly | 41 | 59 | 2.03E-03 | 3.41E-02 | ▼▼ |
|  | Regulation of pyruvate dehydrogenase (PDH) complex | 15 | 35 | 2.79E-03 | 4.33E-02 | ▼▼ |
| **Metabolism of proteins** | Eukaryotic Translation Elongation | 70 | 98 | 1.95E-13 | 4.01E-10 | ▼▼ |
|  | Peptide chain elongation | 67 | 93 | 3.04E-13 | 4.01E-10 | ▼▼ |
|  | Eukaryotic Translation Termination | 72 | 102 | 1.87E-12 | 1.65E-09 | ▼▼ |
|  | Formation of a pool of free 40S subunits | 78 | 102 | 4.46E-10 | 1.68E-07 | ▼▼ |
|  | L13a-mediated translational silencing of Ceruloplasmin expression | 85 | 115 | 8.35E-08 | 1.83E-05 | ▼▼ |
|  | GTP hydrolysis and joining of the 60S ribosomal subunit | 86 | 116 | 1.63E-07 | 3.08E-05 | ▼▼ |
|  | Cap-dependent Translation Initiation | 91 | 125 | 9.79E-07 | 1.52E-04 | ▼▼ |
|  | Eukaryotic Translation Initiation | 91 | 125 | 9.79E-07 | 1.52E-04 | ▼▼ |
|  | Proteasome assembly | 44 | 52 | 9.79E-05 | 5.91E-03 | ▲▲ |
|  | Formation of the ternary complex, and subsequently, the 43S complex | 41 | 53 | 1.04E-04 | 5.93E-03 | ▼▼ |
|  | SRP-dependent cotranslational protein targeting to membrane | 90 | 115 | 1.22E-04 | 6.45E-03 | ▼▼ |
|  | Prefoldin mediated transfer of substrate to CCT/TriC | 24 | 29 | 3.04E-04 | 1.16E-02 | ▲▲ |
|  | Cooperation of Prefoldin and TriC/CCT in actin and tubulin folding | 26 | 37 | 6.62E-04 | 1.80E-02 | ▲▲ |
|  | Mitochondrial protein degradation | 87 | 104 | 9.57E-04 | 2.03E-02 | ▼▼ |
|  | Translation initiation complex formation | 46 | 60 | 1.21E-03 | 2.25E-02 | ▼▼ |
|  | Ribosomal scanning and start codon recognition | 46 | 63 | 1.21E-03 | 2.25E-02 | ▼▼ |
|  | Transport to the Golgi and subsequent modification | 157 | 221 | 1.31E-03 | 2.41E-02 | ▲▲ |
|  | Activation of the mRNA upon binding of the cap-binding complex and eIFs, and subsequent binding to 43S | 47 | 64 | 1.69E-03 | 2.93E-02 | ▼▼ |
|  | Asparagine N-linked glycosylation | 265 | 442 | 2.94E-03 | 4.38E-02 | ▲▲ |
|  | N-glycan antennae elongation in the medial/trans-Golgi | 22 | 47 | 2.99E-03 | 4.38E-02 | ▲▲ |
|  | Formation of tubulin folding intermediates by CCT/TriC | 19 | 30 | 3.53E-03 | 4.83E-02 | ▲▲ |
| **Metabolism of RNA** | Nonsense Mediated Decay (NMD) independent of the Exon Junction Complex (EJC) | 73 | 97 | 3.56E-10 | 1.56E-07 | ▼▼ |
|  | Nonsense-Mediated Decay (NMD) | 92 | 120 | 5.91E-08 | 1.42E-05 | ▼▼ |
|  | Nonsense Mediated Decay (NMD) enhanced by the Exon Junction Complex (EJC) | 92 | 120 | 5.91E-08 | 1.42E-05 | ▼▼ |
|  | rRNA processing | 173 | 241 | 2.20E-04 | 9.73E-03 | ▼▼ |
|  | AUF1 (hnRNP D0) binds and destabilizes mRNA | 37 | 43 | 5.51E-04 | 1.71E-02 | ▲▲ |
|  | Major pathway of rRNA processing in the nucleolus and cytosol | 154 | 185 | 5.72E-04 | 1.75E-02 | ▼▼ |
|  | rRNA processing in the nucleus and cytosol | 163 | 203 | 6.49E-04 | 1.80E-02 | ▼▼ |
| **Programmed cell death** | Activation of caspases through apoptosome-mediated cleavage | 6 | 8 | 1.92E-04 | 8.92E-03 | ▲▲ |
|  | Regulation of activated PAK-2p34 by proteasome mediated degradation | 33 | 37 | 7.54E-04 | 1.89E-02 | ▲▲ |
|  | SMAC (DIABLO) binds to IAPs | 7 | 7 | 8.42E-04 | 1.95E-02 | ▲▲ |
|  | SMAC(DIABLO)-mediated dissociation of IAP:caspase complexes | 7 | 7 | 8.42E-04 | 1.95E-02 | ▲▲ |
|  | SMAC, XIAP-regulated apoptotic response | 7 | 8 | 8.42E-04 | 1.95E-02 | ▲▲ |
|  | Stimulation of the cell death response by PAK-2p34 | 2 | 4 | 1.13E-03 | 2.24E-02 | ▲▲ |
|  | Apoptosis | 145 | 180 | 1.19E-03 | 2.25E-02 | ▲▲ |
|  | Programmed Cell Death | 172 | 225 | 2.12E-03 | 3.52E-02 | ▲▲ |
|  | Caspase activation via Death Receptors in the presence of ligand | 12 | 20 | 2.29E-03 | 3.70E-02 | ▲▲ |
|  | Regulation of Apoptosis | 36 | 42 | 2.91E-03 | 4.38E-02 | ▲▲ |
| **Protein localization** | Peroxisomal protein import | 53 | 67 | 1.20E-04 | 6.45E-03 | ▼▼ |
|  | Protein localization | 138 | 171 | 5.05E-04 | 1.64E-02 | ▼▼ |
| **Signal transduction** | RHO GTPases Activate Formins | 127 | 149 | 1.54E-05 | 1.62E-03 | ▲▲ |
|  | RHO GTPase Effectors | 247 | 325 | 5.14E-05 | 3.87E-03 | ▲▲ |
|  | Regulation of RAS by GAPs | 49 | 58 | 2.58E-04 | 1.10E-02 | ▲▲ |
|  | MET promotes cell motility | 40 | 45 | 4.72E-04 | 1.59E-02 | ▲▲ |
|  | Signaling by Rho GTPases | 595 | 708 | 4.90E-04 | 1.64E-02 | ▲▲ |
|  | MAPK6/MAPK4 signaling | 74 | 93 | 5.94E-04 | 1.75E-02 | ▲▲ |
|  | Signaling by PDGF | 55 | 70 | 5.99E-04 | 1.75E-02 | ▲▲ |
|  | Signaling by Rho GTPases, Miro GTPases and RHOBTB3 | 610 | 724 | 6.08E-04 | 1.75E-02 | ▲▲ |
|  | p38MAPK events | 14 | 19 | 6.33E-04 | 1.77E-02 | ▲▲ |
|  | Hedgehog ligand biogenesis | 45 | 59 | 6.54E-04 | 1.80E-02 | ▲▲ |
|  | Negative regulation of NOTCH4 signaling | 39 | 47 | 7.81E-04 | 1.89E-02 | ▲▲ |
|  | MET activates PTK2 signaling | 29 | 32 | 1.52E-03 | 2.72E-02 | ▲▲ |
|  | RAC1 GTPase cycle | 172 | 191 | 1.66E-03 | 2.93E-02 | ▲▲ |
|  | Signalling to RAS | 19 | 26 | 2.04E-03 | 3.41E-02 | ▲▲ |
|  | RHO GTPases Activate WASPs and WAVEs | 33 | 41 | 2.17E-03 | 3.57E-02 | ▲▲ |
|  | Signalling to ERKs | 32 | 42 | 2.26E-03 | 3.68E-02 | ▲▲ |
|  | RHO GTPase cycle | 407 | 460 | 2.76E-03 | 4.31E-02 | ▲▲ |
|  | PTK6 promotes HIF1A stabilization | 6 | 9 | 3.36E-03 | 4.69E-02 | ▲▲ |
| **Transport of small molecules** | Metal ion SLC transporters | 18 | 31 | 7.76E-04 | 1.89E-02 | ▲▲ |
| **Vesicle-mediated transport** | Golgi-to-ER retrograde transport | 121 | 148 | 9.86E-05 | 5.91E-03 | ▲▲ |
|  | COPI-dependent Golgi-to-ER retrograde traffic | 88 | 107 | 1.03E-04 | 5.93E-03 | ▲▲ |
|  | Gap junction degradation | 11 | 12 | 2.21E-04 | 9.73E-03 | ▲▲ |
|  | Gap junction trafficking | 35 | 52 | 3.01E-04 | 1.16E-02 | ▲▲ |
|  | Formation of annular gap junctions | 10 | 11 | 4.54E-04 | 1.59E-02 | ▲▲ |
|  | Gap junction trafficking and regulation | 37 | 57 | 4.63E-04 | 1.59E-02 | ▲▲ |
|  | Intra-Golgi and retrograde Golgi-to-ER traffic | 185 | 219 | 1.21E-03 | 2.25E-02 | ▲▲ |

**Table S3. Full Reactome enriched pathways of trigone transcriptomic analysis of pBOO vs. NSC bladder tissue.** Pathways are filtered by p-values ≤ 0.005; displayed pathways are based on FDR p-values ≤ 0.05.

| **Categories** | **Pathway name** | **Entities found** | **Entities Total** | **Entities p-Value** | **Entities FDR** | **Expression** |
| --- | --- | --- | --- | --- | --- | --- |
| **Cell cycle** | Mitotic Metaphase and Anaphase | 204 | 237 | 3.26E-05 | 2.97E-03 | ▲▲ |
|  | Mitotic Anaphase | 203 | 236 | 3.62E-05 | 3.20E-03 | ▲▲ |
|  | Separation of Sister Chromatids | 158 | 182 | 4.42E-05 | 3.44E-03 | ▲▲ |
|  | Resolution of Sister Chromatid Cohesion | 113 | 135 | 5.00E-05 | 3.71E-03 | ▲▲ |
|  | The role of GTSE1 in G2/M progression after G2 checkpoint | 52 | 70 | 1.01E-04 | 5.87E-03 | ▲▲ |
|  | Cell Cycle Checkpoints | 234 | 266 | 1.07E-04 | 5.87E-03 | ▲▲ |
|  | EML4 and NUDC in mitotic spindle formation | 103 | 121 | 1.33E-04 | 6.74E-03 | ▲▲ |
|  | Amplification of signal from the kinetochores | 89 | 94 | 1.40E-04 | 6.84E-03 | ▲▲ |
|  | Amplification of signal from unattached kinetochores via a MAD2 inhibitory signal | 89 | 94 | 1.40E-04 | 6.84E-03 | ▲▲ |
|  | Activation of APC/C and APC/C:Cdc20 mediated degradation of mitotic proteins | 58 | 64 | 1.55E-04 | 7.32E-03 | ▲▲ |
|  | Condensation of Prometaphase Chromosomes | 10 | 15 | 1.93E-04 | 7.83E-03 | ▲▲ |
|  | Deposition of new CENPA-containing nucleosomes at the centromere | 36 | 53 | 2.07E-04 | 7.83E-03 | ▲▲ |
|  | Nucleosome assembly | 36 | 53 | 2.07E-04 | 7.83E-03 | ▲▲ |
|  | Synthesis of DNA | 102 | 119 | 2.27E-04 | 8.23E-03 | ▲▲ |
|  | M Phase | 324 | 403 | 2.54E-04 | 8.95E-03 | ▲▲ |
|  | APC/C:Cdc20 mediated degradation of mitotic proteins | 57 | 63 | 2.57E-04 | 8.95E-03 | ▲▲ |
|  | Orc1 removal from chromatin (DNA replication) | 53 | 60 | 2.91E-04 | 9.51E-03 | ▲▲ |
|  | Cell Cycle, Mitotic | 483 | 586 | 3.10E-04 | 9.81E-03 | ▲▲ |
|  | APC:Cdc20 mediated degradation of cell cycle proteins prior to satisfation of the cell cycle checkpoint | 56 | 61 | 4.33E-04 | 1.24E-02 | ▲▲ |
|  | Regulation of mitotic cell cycle | 68 | 79 | 4.60E-04 | 1.30E-02 | ▲▲ |
|  | APC/C-mediated degradation of cell cycle proteins | 68 | 79 | 4.60E-04 | 1.30E-02 | ▲▲ |
|  | Mitotic Spindle Checkpoint | 105 | 111 | 4.86E-04 | 1.33E-02 | ▲▲ |
|  | G2/M Checkpoints | 116 | 142 | 5.57E-04 | 1.48E-02 | ▲▲ |
|  | Regulation of APC/C activators between G1/S and early anaphase | 63 | 70 | 6.55E-04 | 1.61E-02 | ▲▲ |
|  | Cdc20:Phospho-APC/C mediated degradation of Cyclin A | 55 | 60 | 8.07E-04 | 1.89E-02 | ▲▲ |
|  | Ubiquitin Mediated Degradation of Phosphorylated Cdc25A | 34 | 41 | 9.60E-04 | 2.05E-02 | ▲▲ |
|  | p53-Independent DNA Damage Response | 34 | 41 | 9.60E-04 | 2.05E-02 | ▲▲ |
|  | p53-Independent G1/S DNA damage checkpoint | 34 | 41 | 9.60E-04 | 2.05E-02 | ▲▲ |
|  | Condensation of Prophase Chromosomes | 27 | 54 | 9.70E-04 | 2.05E-02 | ▲▲ |
|  | Mitotic Prometaphase | 175 | 212 | 1.03E-03 | 2.09E-02 | ▲▲ |
|  | Switching of origins to a post-replicative state | 73 | 81 | 1.06E-03 | 2.10E-02 | ▲▲ |
|  | Mitotic G1 phase and G1/S transition | 147 | 163 | 1.40E-03 | 2.52E-02 | ▲▲ |
|  | SCF(Skp2)-mediated degradation of p27/p21 | 46 | 52 | 1.50E-03 | 2.64E-02 | ▲▲ |
|  | G1/S Transition | 124 | 140 | 2.01E-03 | 3.15E-02 | ▲▲ |
|  | Cell Cycle | 603 | 722 | 2.04E-03 | 3.16E-02 | ▲▲ |
|  | Ubiquitin-dependent degradation of Cyclin D | 35 | 41 | 2.82E-03 | 3.89E-02 | ▲▲ |
|  | Golgi Cisternae Pericentriolar Stack Reorganization | 13 | 17 | 3.29E-03 | 4.36E-02 | ▲▲ |
|  | Initiation of Nuclear Envelope (NE) Reformation | 20 | 27 | 3.30E-03 | 4.36E-02 | ▲▲ |
|  | SCF-beta-TrCP mediated degradation of Emi1 | 38 | 42 | 3.38E-03 | 4.38E-02 | ▲▲ |
|  | Activation of ATR in response to replication stress | 34 | 39 | 3.84E-03 | 4.79E-02 | ▲▲ |
|  | Autodegradation of the E3 ubiquitin ligase COP1 | 35 | 41 | 4.13E-03 | 4.99E-02 | ▲▲ |
| **Cell-cell communication** | Type I hemidesmosome assembly | 9 | 11 | 4.13E-03 | 4.99E-02 | ▲▲ |
| **Cellular responses to stimuli** | Response of EIF2AK4 (GCN2) to amino acid deficiency | 86 | 111 | 5.60E-04 | 1.48E-02 | ▼▼ |
|  | Oxygen-dependent proline hydroxylation of Hypoxia-inducible Factor Alpha | 47 | 59 | 9.98E-04 | 2.08E-02 | ▲▲ |
|  | Heme signaling | 47 | 70 | 1.02E-03 | 2.09E-02 | ▼▼ |
|  | Unfolded Protein Response (UPR) | 145 | 156 | 2.44E-03 | 3.55E-02 | ▲▲ |
|  | Turbulent (oscillatory, disturbed) flow shear stress activates signaling by PIEZO1 and integrins in endothelial cells | 31 | 42 | 3.57E-03 | 4.56E-02 | ▲▲ |
| **Circadian clock** | Expression of BMAL (ARNTL), CLOCK, and NPAS2 | 36 | 46 | 1.52E-03 | 2.66E-02 | ▼▼ |
| **Developmental biology** | Somitogenesis | 40 | 53 | 3.25E-05 | 2.97E-03 | ▲▲ |
|  | EPH-Ephrin signaling | 86 | 102 | 3.94E-04 | 1.17E-02 | ▲▲ |
|  | EPHB-mediated forward signaling | 40 | 52 | 6.97E-04 | 1.66E-02 | ▲▲ |
| **Disease** | Viral mRNA Translation | 68 | 101 | 1.43E-06 | 4.72E-04 | ▼▼ |
|  | Leishmania phagocytosis | 52 | 157 | 1.55E-05 | 1.86E-03 | ▲▲ |
|  | Parasite infection | 52 | 157 | 1.55E-05 | 1.86E-03 | ▲▲ |
|  | FCGR3A-mediated phagocytosis | 52 | 157 | 1.55E-05 | 1.86E-03 | ▲▲ |
|  | Maturation of spike protein | 37 | 48 | 4.29E-05 | 3.44E-03 | ▲▲ |
|  | Diseases of Immune System | 27 | 47 | 5.39E-05 | 3.86E-03 | ▲▲ |
|  | Diseases associated with the TLR signaling cascade | 27 | 47 | 5.39E-05 | 3.86E-03 | ▲▲ |
|  | CD163 mediating an anti-inflammatory response | 10 | 14 | 9.62E-05 | 5.79E-03 | ▲▲ |
|  | Translation of Structural Proteins | 54 | 85 | 1.04E-04 | 5.87E-03 | ▲▲ |
|  | Late SARS-CoV-2 Infection Events | 65 | 102 | 1.20E-04 | 6.34E-03 | ▲▲ |
|  | Diseases of branched-chain amino acid catabolism | 13 | 32 | 1.50E-04 | 7.24E-03 | ▼▼ |
|  | Hh mutants are degraded by ERAD | 39 | 48 | 3.00E-04 | 9.67E-03 | ▲▲ |
|  | Hh mutants abrogate ligand secretion | 42 | 51 | 4.04E-04 | 1.17E-02 | ▲▲ |
|  | Nucleotide salvage | 18 | 69 | 4.75E-04 | 1.32E-02 | ▲▲ |
|  | SARS-CoV-1 modulates host translation machinery | 27 | 40 | 5.73E-04 | 1.49E-02 | ▼▼ |
|  | Microbial modulation of RIPK1-mediated regulated necrosis | 4 | 10 | 9.21E-04 | 2.05E-02 | ▲▲ |
|  | Diseases of programmed cell death | 58 | 97 | 1.03E-03 | 2.09E-02 | ▲▲ |
|  | Defective CFTR causes cystic fibrosis | 43 | 53 | 1.33E-03 | 2.45E-02 | ▲▲ |
|  | Defective RIPK1-mediated regulated necrosis | 6 | 13 | 2.12E-03 | 3.24E-02 | ▲▲ |
|  | Vif-mediated degradation of APOBEC3G | 36 | 43 | 2.17E-03 | 3.30E-02 | ▲▲ |
|  | MyD88 deficiency (TLR2/4) | 12 | 26 | 2.92E-03 | 4.00E-02 | ▲▲ |
|  | Influenza Viral RNA Transcription and Replication | 115 | 154 | 3.18E-03 | 4.31E-02 | ▼▼ |
|  | IRAK4 deficiency (TLR2/4) | 13 | 27 | 3.78E-03 | 4.77E-02 | ▲▲ |
| **DNA replication** | DNA Replication Pre-Initiation | 96 | 121 | 3.42E-04 | 1.06E-02 | ▲▲ |
|  | DNA Replication | 131 | 171 | 6.64E-04 | 1.61E-02 | ▲▲ |
|  | Assembly of the pre-replicative complex | 80 | 103 | 8.70E-04 | 1.97E-02 | ▲▲ |
|  | CDK-mediated phosphorylation and removal of Cdc6 (Cell cycle) | 55 | 62 | 1.74E-03 | 2.88E-02 | ▲▲ |
|  | (celll cycl | 31 | 38 | 2.27E-03 | 3.37E-02 | ▲▲ |
|  | Unwinding of DNA (Cell Cycle) | 11 | 12 | 2.43E-03 | 3.55E-02 | ▲▲ |
|  | Activation of the pre-replicative complex (Cell cycle) | 31 | 36 | 3.32E-03 | 4.36E-02 | ▲▲ |
| **Extracellular matrix organization** | Collagen formation | 81 | 104 | 4.65E-06 | 9.47E-04 | ▲▲ |
|  | Assembly of collagen fibrils and other multimeric structures | 55 | 67 | 1.52E-05 | 1.86E-03 | ▲▲ |
|  | Degradation of the extracellular matrix | 111 | 148 | 1.69E-04 | 7.44E-03 | ▲▲ |
|  | Collagen degradation | 53 | 69 | 1.84E-04 | 7.83E-03 | ▲▲ |
|  | Syndecan interactions | 26 | 29 | 1.96E-04 | 7.83E-03 | ▲▲ |
|  | Integrin cell surface interactions | 72 | 86 | 1.06E-03 | 2.10E-02 | ▲▲ |
|  | Collagen biosynthesis and modifying enzymes | 59 | 76 | 1.16E-03 | 2.22E-02 | ▲▲ |
|  | Activation of Matrix Metalloproteinases | 19 | 35 | 2.03E-03 | 3.16E-02 | ▲▲ |
|  | Extracellular matrix organization | 271 | 351 | 3.60E-03 | 4.56E-02 | ▲▲ |
| **Gene expression (Transcription)** | Regulation of FOXO transcriptional activity by acetylation | 9 | 16 | 1.58E-04 | 7.32E-03 | ▼▼ |
|  | Regulation of RUNX3 expression and activity | 41 | 47 | 7.42E-04 | 1.75E-02 | ▲▲ |
|  | RUNX2 regulates genes involved in differentiation of myeloid cells | 6 | 6 | 9.34E-04 | 2.05E-02 | ▲▲ |
|  | FOXO-mediated transcription of oxidative stress, metabolic and neuronal genes | 30 | 49 | 1.12E-03 | 2.19E-02 | ▼▼ |
|  | RUNX3 regulates RUNX1-mediated transcription | 4 | 4 | 3.34E-03 | 4.36E-02 | ▲▲ |
| **Hemostasis** | Dissolution of Fibrin Clot | 10 | 14 | 2.68E-06 | 7.10E-04 | ▲▲ |
|  | cGMP effects | 14 | 18 | 3.58E-04 | 1.09E-02 | ▼▼ |
|  | GPVI-mediated activation cascade | 31 | 43 | 6.56E-04 | 1.61E-02 | ▲▲ |
|  | Platelet Adhesion to exposed collagen | 14 | 17 | 8.41E-04 | 1.92E-02 | ▲▲ |
|  | Nitric oxide stimulates guanylate cyclase | 20 | 35 | 2.39E-03 | 3.52E-02 | ▼▼ |
| **Immune system** | ER-Phagosome pathway | 66 | 98 | 8.80E-08 | 1.69E-04 | ▲▲ |
|  | Antigen processing-Cross presentation | 80 | 120 | 1.28E-07 | 1.69E-04 | ▲▲ |
|  | Neutrophil degranulation | 368 | 478 | 9.81E-07 | 3.81E-04 | ▲▲ |
|  | Modulation of host responses by IFN-stimulated genes | 18 | 22 | 4.45E-06 | 9.47E-04 | ▲▲ |
|  | Interferon alpha/beta signaling | 87 | 129 | 6.62E-06 | 1.25E-03 | ▲▲ |
|  | Innate Immune System | 789 | 1,334 | 1.49E-05 | 1.86E-03 | ▲▲ |
|  | Cross-presentation of soluble exogenous antigens (endosomes) | 37 | 46 | 2.38E-05 | 2.52E-03 | ▲▲ |
|  | Cytokine Signaling in Immune system | 834 | 1,095 | 3.23E-05 | 2.97E-03 | ▲▲ |
|  | Signaling by Interleukins | 515 | 646 | 4.11E-05 | 3.44E-03 | ▲▲ |
|  | Regulation of actin dynamics for phagocytic cup formation | 52 | 158 | 4.36E-05 | 3.44E-03 | ▲▲ |
|  | Antigen processing: Ub, ATP-independent proteasomal degradation | 17 | 21 | 1.07E-04 | 5.87E-03 | ▲▲ |
|  | Fcgamma receptor (FCGR) dependent phagocytosis | 77 | 193 | 1.09E-04 | 5.87E-03 | ▲▲ |
|  | Interleukin-12 family signaling | 72 | 96 | 1.23E-04 | 6.38E-03 | ▲▲ |
|  | Immune System | 1791 | 2,664 | 1.62E-04 | 7.40E-03 | ▲▲ |
|  | ZBP1(DAI) mediated induction of type I IFNs | 21 | 23 | 1.67E-04 | 7.44E-03 | ▲▲ |
|  | Interferon Signaling | 283 | 404 | 2.00E-04 | 7.83E-03 | ▲▲ |
|  | Interleukin-10 signaling | 67 | 86 | 2.21E-04 | 8.13E-03 | ▲▲ |
|  | Antigen Presentation: Folding, assembly and peptide loading of class I MHC | 22 | 41 | 2.74E-04 | 9.29E-03 | ▲▲ |
|  | TCR signaling | 91 | 122 | 2.86E-04 | 9.47E-03 | ▲▲ |
|  | Interleukin-1 family signaling | 131 | 170 | 3.12E-04 | 9.81E-03 | ▲▲ |
|  | MHC class II antigen presentation | 104 | 137 | 3.90E-04 | 1.17E-02 | ▲▲ |
|  | Signaling by the B Cell Receptor (BCR) | 91 | 176 | 5.91E-04 | 1.51E-02 | ▲▲ |
|  | Gene and protein expression by JAK-STAT signaling after Interleukin-12 stimulation | 57 | 73 | 5.95E-04 | 1.51E-02 | ▲▲ |
|  | NF-kB activation through FADD/RIP-1 pathway mediated by caspase-8 and -10 | 12 | 14 | 6.14E-04 | 1.54E-02 | ▲▲ |
|  | RIP-mediated NFkB activation via ZBP1 | 17 | 19 | 8.22E-04 | 1.91E-02 | ▲▲ |
|  | Interleukin-12 signaling | 63 | 84 | 9.20E-04 | 2.05E-02 | ▲▲ |
|  | Activation of NF-kappaB in B cells | 50 | 59 | 9.64E-04 | 2.05E-02 | ▲▲ |
|  | TRIF-mediated programmed cell death | 8 | 10 | 1.01E-03 | 2.09E-02 | ▲▲ |
|  | Interleukin-1 signaling | 90 | 112 | 1.30E-03 | 2.41E-02 | ▲▲ |
|  | Toll-like Receptor Cascades | 158 | 210 | 1.45E-03 | 2.59E-02 | ▲▲ |
|  | Interferon gamma signaling | 111 | 177 | 1.53E-03 | 2.67E-02 | ▲▲ |
|  | Downstream signaling events of B Cell Receptor (BCR) | 64 | 78 | 1.55E-03 | 2.68E-02 | ▲▲ |
|  | PKR-mediated signaling | 60 | 88 | 1.57E-03 | 2.68E-02 | ▲▲ |
|  | Trafficking and processing of endosomal TLR | 13 | 16 | 1.61E-03 | 2.74E-02 | ▲▲ |
|  | TNFR2 non-canonical NF-kB pathway | 77 | 91 | 1.65E-03 | 2.79E-02 | ▲▲ |
|  | Dectin-1 mediated noncanonical NF-kB signaling | 45 | 53 | 1.67E-03 | 2.80E-02 | ▲▲ |
|  | NIK-->noncanonical NF-kB signaling | 42 | 48 | 2.23E-03 | 3.34E-02 | ▲▲ |
|  | Downstream TCR signaling | 70 | 99 | 2.55E-03 | 3.61E-02 | ▲▲ |
|  | CLEC7A (Dectin-1) signaling | 80 | 107 | 2.57E-03 | 3.62E-02 | ▲▲ |
|  | C-type lectin receptors (CLRs) | 102 | 190 | 2.95E-03 | 4.02E-02 | ▲▲ |
| **Metabolism** | Aerobic respiration and respiratory electron transport | 231 | 361 | 1.01E-06 | 3.81E-04 | ▼▼ |
|  | Selenocysteine synthesis | 71 | 108 | 2.27E-06 | 6.66E-04 | ▼▼ |
|  | Branched-chain amino acid catabolism | 21 | 59 | 8.85E-06 | 1.56E-03 | ▼▼ |
|  | Respiratory electron transport | 142 | 197 | 1.45E-05 | 1.86E-03 | ▼▼ |
|  | Metabolism of polyamines | 39 | 67 | 2.23E-05 | 2.46E-03 | ▲▲ |
|  | Regulation of ornithine decarboxylase (ODC) | 33 | 38 | 7.31E-05 | 4.72E-03 | ▲▲ |
|  | Citric acid cycle (TCA cycle) | 34 | 83 | 6.16E-04 | 1.54E-02 | ▼▼ |
|  | Mitochondrial Fatty Acid Beta-Oxidation | 31 | 105 | 6.74E-04 | 1.62E-02 | ▼▼ |
|  | Regulation of pyruvate dehydrogenase (PDH) complex | 15 | 35 | 8.40E-04 | 1.92E-02 | ▼▼ |
|  | Complex I biogenesis | 60 | 71 | 1.25E-03 | 2.37E-02 | ▼▼ |
|  | Metabolism of ingested H2SeO4 and H2SeO3 into H2Se | 4 | 22 | 1.28E-03 | 2.39E-02 | ▲▲ |
|  | Pyrimidine salvage | 7 | 34 | 2.08E-03 | 3.20E-02 | ▲▲ |
|  | Pyruvate metabolism | 43 | 96 | 2.23E-03 | 3.34E-02 | ▼▼ |
|  | Hyaluronan metabolism | 16 | 23 | 2.37E-03 | 3.51E-02 | ▲▲ |
|  | Transport and synthesis of PAPS | 6 | 13 | 3.19E-03 | 4.31E-02 | ▲▲ |
|  | Complex IV assembly | 41 | 59 | 3.29E-03 | 4.36E-02 | ▼▼ |
|  | Selenoamino acid metabolism | 93 | 178 | 3.46E-03 | 4.46E-02 | ▼▼ |
|  | Maturation of TCA enzymes and regulation of TCA cycle | 19 | 47 | 3.47E-03 | 4.46E-02 | ▼▼ |
|  | Regulation of pyruvate metabolism | 33 | 54 | 3.58E-03 | 4.56E-02 | ▼▼ |
| **Metabolism of proteins** | Eukaryotic Translation Elongation | 70 | 98 | 2.61E-07 | 2.30E-04 | ▼▼ |
|  | Peptide chain elongation | 67 | 93 | 4.75E-07 | 3.14E-04 | ▼▼ |
|  | Eukaryotic Translation Termination | 72 | 102 | 9.13E-07 | 3.81E-04 | ▼▼ |
|  | Formation of a pool of free 40S subunits | 78 | 102 | 3.03E-06 | 7.30E-04 | ▼▼ |
|  | L13a-mediated translational silencing of Ceruloplasmin expression | 85 | 115 | 1.38E-05 | 1.86E-03 | ▼▼ |
|  | GTP hydrolysis and joining of the 60S ribosomal subunit | 86 | 116 | 1.73E-05 | 1.99E-03 | ▼▼ |
|  | Cap-dependent Translation Initiation | 91 | 125 | 6.30E-05 | 4.17E-03 | ▼▼ |
|  | Eukaryotic Translation Initiation | 91 | 125 | 6.30E-05 | 4.17E-03 | ▼▼ |
|  | O-linked glycosylation of mucins | 41 | 73 | 2.34E-04 | 8.35E-03 | ▲▲ |
|  | Proteasome assembly | 44 | 52 | 2.65E-04 | 9.12E-03 | ▲▲ |
|  | Asparagine N-linked glycosylation | 269 | 442 | 2.82E-04 | 9.44E-03 | ▲▲ |
|  | N-glycan antennae elongation in the medial/trans-Golgi | 22 | 47 | 3.48E-04 | 1.07E-02 | ▲▲ |
|  | Mitochondrial protein degradation | 87 | 104 | 1.30E-03 | 2.41E-02 | ▼▼ |
|  | Transport to the Golgi and subsequent modification | 159 | 221 | 1.40E-03 | 2.52E-02 | ▲▲ |
|  | N-Glycan antennae elongation | 14 | 20 | 1.57E-03 | 2.68E-02 | ▲▲ |
|  | Synthesis of substrates in N-glycan biosythesis | 58 | 146 | 2.77E-03 | 3.85E-02 | ▲▲ |
|  | Regulation of Insulin-like Growth Factor (IGF) transport and uptake by Insulin-like Growth Factor Binding Proteins (IGFBPs) | 96 | 127 | 3.26E-03 | 4.36E-02 | ▲▲ |
|  | Formation of the ternary complex, and subsequently, the 43S complex | 41 | 53 | 3.35E-03 | 4.36E-02 | ▼▼ |
|  | Translation initiation complex formation | 46 | 60 | 4.10E-03 | 4.99E-02 | ▼▼ |
|  | Ribosomal scanning and start codon recognition | 46 | 63 | 4.10E-03 | 4.99E-02 | ▼▼ |
| **Metabolism of RNA** | Nonsense Mediated Decay (NMD) independent of the Exon Junction Complex (EJC) | 73 | 97 | 2.50E-05 | 2.55E-03 | ▼▼ |
|  | Nonsense-Mediated Decay (NMD) | 92 | 120 | 1.94E-04 | 7.83E-03 | ▼▼ |
|  | Nonsense Mediated Decay (NMD) enhanced by the Exon Junction Complex (EJC) | 92 | 120 | 1.94E-04 | 7.83E-03 | ▼▼ |
|  | AUF1 (hnRNP D0) binds and destabilizes mRNA | 37 | 43 | 5.02E-04 | 1.36E-02 | ▲▲ |
| **Neuronal system** | Synaptic adhesion-like molecules | 19 | 23 | 1.91E-03 | 3.02E-02 | ▼▼ |
| **Organelle biogenesis** | Transcriptional activation of mitochondrial biogenesis | 67 | 89 | 1.49E-03 | 2.64E-02 | ▼▼ |
|  | Mitochondrial biogenesis | 103 | 131 | 3.83E-03 | 4.79E-02 | ▼▼ |
| **Programmed cell death** | Apoptotic factor-mediated response | 17 | 23 | 1.76E-03 | 2.88E-02 | ▲▲ |
|  | Cytochrome c-mediated apoptotic response | 12 | 15 | 1.76E-03 | 2.88E-02 | ▲▲ |
|  | Apoptosis | 147 | 180 | 1.79E-03 | 2.90E-02 | ▲▲ |
|  | Programmed Cell Death | 174 | 225 | 2.22E-03 | 3.34E-02 | ▲▲ |
|  | Regulation of activated PAK-2p34 by proteasome mediated degradation | 33 | 37 | 9.90E-04 | 2.08E-02 | ▲▲ |
|  | Caspase activation via Death Receptors in the presence of ligand | 12 | 20 | 1.06E-03 | 2.10E-02 | ▲▲ |
|  | Activation of caspases through apoptosome-mediated cleavage | 6 | 8 | 1.35E-03 | 2.47E-02 | ▲▲ |
|  | SMAC (DIABLO) binds to IAPs | 7 | 7 | 2.53E-03 | 3.60E-02 | ▲▲ |
|  | SMAC(DIABLO)-mediated dissociation of IAP:caspase complexes | 7 | 7 | 2.53E-03 | 3.60E-02 | ▲▲ |
|  | SMAC, XIAP-regulated apoptotic response | 7 | 8 | 2.53E-03 | 3.60E-02 | ▲▲ |
|  | Regulation of Apoptosis | 36 | 42 | 4.04E-03 | 4.97E-02 | ▲▲ |
| **Sensory perception** | Acetylcholine inhibits contraction of outer hair cells | 3 | 8 | 1.86E-03 | 2.97E-02 | ▼▼ |
| **Signal transduction** | RHO GTPases Activate Formins | 126 | 149 | 8.05E-05 | 4.95E-03 | ▲▲ |
|  | Hedgehog ligand biogenesis | 45 | 59 | 2.02E-04 | 7.83E-03 | ▲▲ |
|  | RHO GTPase Effectors | 247 | 325 | 3.97E-04 | 1.17E-02 | ▲▲ |
|  | Regulation of RAS by GAPs | 49 | 58 | 4.88E-04 | 1.33E-02 | ▲▲ |
|  | p38MAPK events | 14 | 19 | 1.08E-03 | 2.13E-02 | ▲▲ |
|  | Negative regulation of NOTCH4 signaling | 39 | 47 | 1.73E-03 | 2.88E-02 | ▲▲ |
|  | Signalling to RAS | 19 | 26 | 1.86E-03 | 2.97E-02 | ▲▲ |
|  | MAPK6/MAPK4 signaling | 72 | 93 | 2.52E-03 | 3.60E-02 | ▲▲ |
|  | Signaling by PDGF | 56 | 70 | 2.68E-03 | 3.75E-02 | ▲▲ |
|  | RHO GTPases Activate WASPs and WAVEs | 33 | 41 | 2.82E-03 | 3.89E-02 | ▲▲ |
|  | RAC1 GTPase cycle | 173 | 191 | 3.85E-03 | 4.79E-02 | ▲▲ |
|  | Signaling by Rho GTPases | 597 | 708 | 3.95E-03 | 4.88E-02 | ▲▲ |
| **Transport of small molecules** | Metal ion SLC transporters | 18 | 31 | 2.15E-04 | 8.00E-03 | ▲▲ |
|  | Zinc transporters | 11 | 16 | 1.78E-03 | 2.89E-02 | ▲▲ |
|  | Zinc influx into cells by the SLC39 gene family | 7 | 11 | 1.92E-03 | 3.02E-02 | ▲▲ |
| **Vesicle-mediated transport** | COPI-dependent Golgi-to-ER retrograde traffic | 87 | 107 | 6.02E-05 | 4.17E-03 | ▲▲ |
|  | Gap junction degradation | 11 | 12 | 7.89E-05 | 4.95E-03 | ▲▲ |
|  | Formation of annular gap junctions | 10 | 11 | 1.73E-04 | 7.51E-03 | ▲▲ |
|  | Gap junction trafficking | 34 | 52 | 5.70E-04 | 1.49E-02 | ▲▲ |
|  | Gap junction trafficking and regulation | 36 | 57 | 1.14E-03 | 2.20E-02 | ▲▲ |
|  | Golgi-to-ER retrograde transport | 120 | 148 | 1.18E-03 | 2.25E-02 | ▲▲ |

**Table S4. Full Reactome enriched pathways of dome transcriptomic analysis of obstructed vs. NSC bladder tissue.** Pathways are filtered by p-values ≤ 0.005; displayed pathways are based on FDR p-values ≤ 0.05.

| **Categories** | **Pathway name** | **Entities found** | **Entities Total** | **Entities p-Value** | **Entities FDR** | **Expression** |
| --- | --- | --- | --- | --- | --- | --- |
| **Disease** | Viral mRNA Translation | 68 | 101 | 6.29E-06 | 2.77E-03 | ▼▼ |
|  | SARS-CoV-1 modulates host translation machinery | 27 | 40 | 2.75E-05 | 1.02E-02 | ▼▼ |
| **Immune System** | Modulation of host responses by IFN-stimulated genes | 18 | 22 | 4.64E-08 | 1.23E-04 | ▲▲ |
|  | Interferon alpha/beta signaling | 83 | 129 | 8.69E-05 | 2.25E-02 | ▲▲ |
|  | Regulation of Complement cascade | 27 | 139 | 1.32E-04 | 2.68E-02 | ▼▼ |
| **Metabolism** | Selenocysteine synthesis | 71 | 108 | 5.14E-06 | 2.72E-03 | ▼▼ |
|  | Phase I - Functionalization of compounds | 63 | 297 | 5.10E-05 | 1.50E-02 | ▼▼ |
|  | Biological oxidations | 133 | 551 | 1.02E-04 | 2.25E-02 | ▼▼ |
|  | Cytochrome P450 - arranged by substrate type | 32 | 185 | 1.80E-04 | 3.40E-02 | ▼▼ |
|  | Synthesis of epoxy (EET) and dihydroxyeicosatrienoic acids (DHET) | 3 | 23 | 2.26E-04 | 3.98E-02 | ▼▼ |
| **Metabolism of proteins** | Eukaryotic Translation Elongation | 70 | 98 | 1.35E-06 | 1.20E-03 | ▼▼ |
|  | Peptide chain elongation | 67 | 93 | 1.36E-06 | 1.20E-03 | ▼▼ |
|  | Eukaryotic Translation Termination | 72 | 102 | 2.21E-06 | 1.46E-03 | ▼▼ |
|  | Formation of a pool of free 40S subunits | 78 | 102 | 1.02E-04 | 2.25E-02 | ▼▼ |
| **Metabolism of RNA** | Nonsense Mediated Decay (NMD) independent of the Exon Junction Complex (EJC) | 73 | 97 | 3.09E-05 | 1.02E-02 | ▼▼ |
